# Harnessing spatiotemporal melatonin delivery from engineered platforms for targeted microenvironment remodeling in peripheral neuropathy

**DOI:** 10.1016/j.mtbio.2026.103144

**Published:** 2026-04-22

**Authors:** Mouyuan Sun, Xuankai Fan, Yaxian Luo, Luying Qin, Shuangyang Li, Jingyu Zhang, Zhixu He, Lianjie Peng, Tao Qiu, Tian Zhang, Huiming Wang, Mengfei Yu

**Affiliations:** Stomatology Hospital, School of Stomatology, Zhejiang University School of Medicine, Zhejiang Provincial Clinical Research Center for Oral Diseases, Zhejiang Key Laboratory of Oral Biomedical, Hangzhou, 310000, China

**Keywords:** Peripheral neuropathy, Inflammation and oxidative stress, Melatonin, Tissue engineering, Circadian rhythm

## Abstract

Peripheral neuropathy, a leading contributor to global disability, arises from persistent neuroinflammation and oxidative stress that progressively dismantle neural support structures. Notably, its pathophysiology is intricately modulated by circadian rhythms. In this context, the endogenous hormone melatonin offers a compelling therapeutic opportunity, given its capacity to orchestrate protective responses across diverse neural cell types. Despite this potential, clinical translation is severely constrained by its suboptimal pharmacokinetic profile. This limitation creates an imperative for innovative biomaterial-based interventions. This review bridges mechanistic insight with engineering design by detailing strategies to achieve spatiotemporally controlled melatonin release through tailored material composition, structural design, and degradation behavior. For traumatic nerve injuries requiring guided regeneration, we examine the utility of implantable conduits. In addressing diffuse neuropathies, we further propose translating these core design principles toward minimally invasive platforms, including injectable hydrogels, microneedles, nanocarriers, and localized depots. The review culminates in a translational roadmap that outlines critical phases for clinical adoption. These phases encompass platform optimization, formulation standardization, rigorous validation of release kinetics, navigation of regulatory pathways for combination products, and the ultimate demonstration of clinical relevance.

## Highlights

• Network meta-analyses reveal inconsistent clinical efficacy across current peripheral neuropathy interventions.
• Advances a circadian-aware, stage-specific spatiotemporal concentration-gradient concept for local melatonin administration.
• Distills transferable tissue-engineering design logic from cross-tissue precedents to guide materials, architecture, and delivery format selection for peripheral neuropathy.
• Dissects current challenges and bottlenecks for clinical translation of melatonin-enabled tissue-engineered systems.

## Graphical abstract

**Figure:**
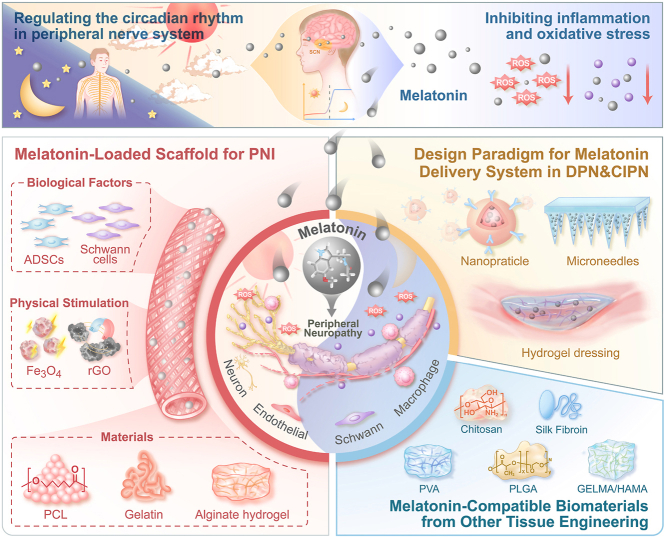

**Table:** List of abbreviations

Abbreviation	Full term
PN	Peripheral neuropathy
PNR	Peripheral nerve repair
PNI	Peripheral nerve injury
DPN	Diabetic peripheral neuropathy
CIPN	Chemotherapy-induced peripheral neuropathy
ROS	Reactive oxygen species
IL-1β	Interleukin-1 beta
TNF-α	Tumor necrosis factor-alpha
NaV1.7	Voltage-gated sodium channel 1.7
IL-6	Interleukin-6
TRPA1	transient receptor potential ankyrin 1
CaV3.2	voltage-gated calcium channel 3.2
SUCRA	Surface under the cumulative ranking curve
WoSCC	Web of Science Core Collection
MLS	Melatonin-loaded scaffold(s)
AANAT	Arylalkylamine N-acetyltransferase
PCL	Polycaprolactone
rGO	Reduced graphene oxide
GelMA	Gelatin Methacryloyl
ECM	Extracellular matrix
DRG	Dorsal root ganglion
3D	Three-dimensional
4D	Four-dimensional
5D	Five-dimensional

## 1 Introduction

Peripheral neuropathy (PN) encompasses a spectrum of peripheral nerve disorders manifesting as sensorimotor impairment, neuropathic pain, enduring disability, and substantial socioeconomic burden [1]. With a global adult prevalence of 7%, representing over 200 million individuals, PN poses a major public health challenge [2,3]. Despite clinical stratification into traumatic and non-traumatic subtypes, both converge on a shared pathological niche defined by neuroinflammation and oxidative stress, microenvironments inherently hostile to neural homeostasis and regeneration [4,5]. Circadian rhythms further entrain disease dynamics, modulating pain periodicity and repair capacity [6,7]. Current protocols remain dichotomous: surgical reconstruction for transected nerves and systemic analgesics for non-traumatic neuropathies [8,9]. Neither regimen targets the core immuno-redox disequilibrium nor leverages circadian gating. This therapeutic void mandates engineering-driven strategies that simultaneously rectify inflammatory-oxidative cascades and integrate circadian timing.

Melatonin, a nocturnally secreted indoleamine orchestrated by the circadian machinery, serves as both a chronobiotic stabilizer and a pleiotropic effector in peripheral nerve homeostasis [10]. In neuropathic microenvironments, it represses pro-inflammatory polarization, neutralizes oxidative insults, and modulates macrophages, Schwann cells, endothelial cells, and neurons [[11], [12], [13], [14]]. Among the diverse bioactive molecules and engineering-derived therapeutic cues currently explored for PN, melatonin distinguishes itself through a truly unique convergence of several favorable attributes. Its endogenous origin minimizes potential immunogenicity risks, while its capacity to engage multiple mechanistic pathways enables the coordinated and dynamic modulation of the injury microenvironment. Together, these features position melatonin as a highly attractive and promising candidate for therapeutic reprogramming in PN [[15], [16], [17]]. Yet its translation is obstructed by poor pharmacokinetics and a narrow, dose-dependent therapeutic window, collectively preventing the sustained, lesion-compartmentalized exposure essential for peripheral nerve repair (PNR) [18]. Overcoming this barrier requires engineered delivery systems capable of translating melatonin's systemic pleiotropy into localized, temporally programmable bioavailability.

Biomaterial-based delivery platforms constitute a powerful solution for spatiotemporally governed melatonin release. Through rational integration with regenerative matrices, these constructs afford systematic control over elution profiles [19]. Preclinical studies confirm that melatonin-laden natural and synthetic polymer scaffolds elicit pronounced PNR efficacy [20,21]. While current PN research centers chiefly on nerve conduits for traumatic injury, alternative paradigms from other chronic conditions, including injectable hydrogels and in situ depots, furnish directly adaptable design solutions for non-traumatic PN, where spatially diffuse pathologies demand distinct therapeutic architectures. By transposing melatonin's pleiotropic actions into precisely titrated local concentrations, these engineering solutions enable active modulation of the diseased neural niche across diverse neuropathic presentations.

This review articulates a circadian-integrated, multitarget therapeutic framework centered on melatonin and its engineered delivery. It consolidates shared pathological hallmarks and therapeutic deficits across PN subtypes, alongside melatonin's cell-specific modulatory circuitry within the nerve microenvironment. This synthesis underscores the critical dependency on delivery technologies that safeguard melatonin's spatiotemporal bioactivity. Particular scrutiny is directed toward biomaterial strategies architected for controlled, lesion-localized melatonin release. The analytical scope stratifies conduit-based scaffolds for traumatic repair and adaptive modalities, including injectable hydrogels and localized depots, engineered for non-traumatic PN, where lesion diffusivity and chronic evolution impose distinct structural and dosing specifications that mandate spatiotemporally programmable delivery. Collectively, these insights converge into a translational design matrix that distills material determinants, architectural parameters, release programming logic, and clinical integration pathways, furnishing core engineering principles for next-generation melatonin-eluting therapeutic systems.

## 2 Pathogenesis of PN and the rationale for engineering interventions

The substantial and growing public health burden of PN arises from its profound clinical and socioeconomic consequences. Traumatic peripheral nerve injury (PNI) afflicts an estimated five million individuals annually [22]; among non-traumatic forms, diabetic peripheral neuropathy (DPN) accounts for over 200 million patients globally [23,24]. Chemotherapy-induced peripheral neuropathy (CIPN) is an increasingly prevalent complication, with up to 15 million annual patients at risk by 2040 [25,26]. Despite distinct etiologies, PNI, DPN, and CIPN converge on shared pathological hallmarks: oxidative stress and neuroinflammation act as common drivers of neural injury, while circadian disruption modulates disease dynamics and repair capacity [[4], [5], [6]]. This section synthesizes these core mechanisms and critically examines the efficacy, translational gaps, and inherent limitations of contemporary therapeutic strategies.

### 2.1 Pathogenesis of PN

**Fig. 1:**
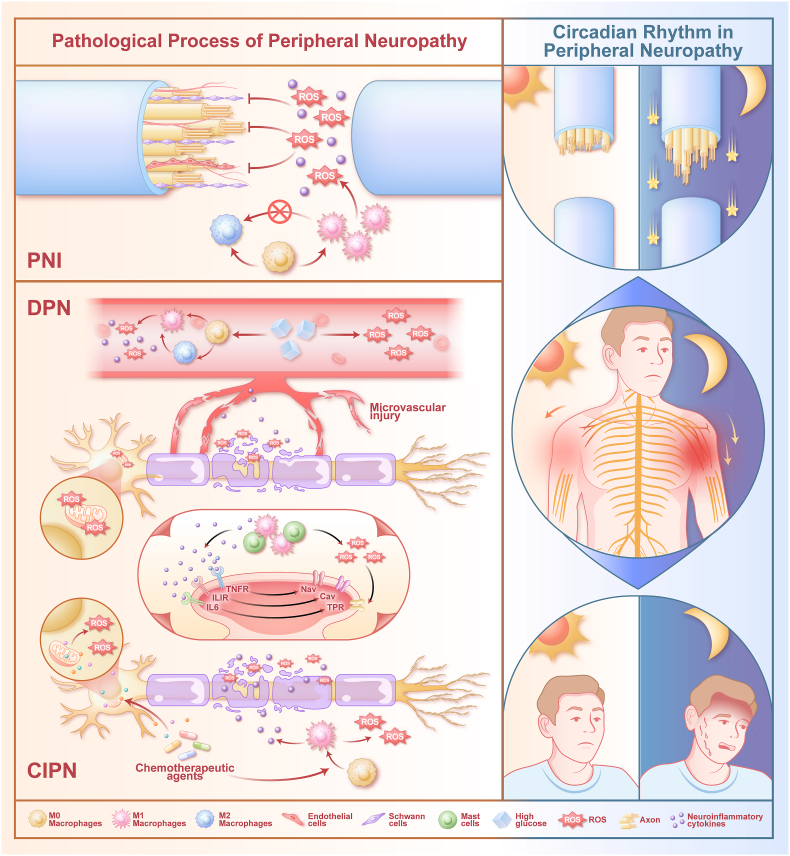
Schematic overview of the shared and distinct pathological processes underlying peripheral neuropathy (PN), including peripheral nerve injury (PNI), diabetic peripheral neuropathy (DPN), and Chemotherapy-induced peripheral neuropathy (CIPN). Across these conditions, neuroinflammation, oxidative stress, immune cell activation, and microvascular dysfunction converge to disrupt axonal integrity and sensory signaling. The right panel highlights circadian regulation of peripheral neuropathy, characterized by accelerated axonal regeneration and enhanced pain sensitivity during the night, which modulates disease progression and symptom severity.

The progressive disintegration of multicellular homeostasis within the peripheral nerve microenvironment constitutes a central pathogenic driver of PN. Aberrant inflammatory signaling and oxidative disequilibrium disseminate across neuronal, glial, immune, and endothelial compartments, collectively fueling tissue degeneration and stifling regenerative programs [27]. Entrained upon these core pathological circuits, circadian control systems exert profound influence over both endogenous repair capacity and the temporal patterning of neuropathic pain [6,28]. This section consolidates these principal pathological pillars, namely inflammatory activation, redox dyshomeostasis, and circadian modulation, as foundational targets for microenvironmental engineering (Fig. 1).

#### 2.1.1 Multicellular injury cascade orchestrated by inflammation and oxidative stress

Peripheral nerve regeneration proceeds through spatially and temporally defined phases of tissue dismantling and reconstruction. Wallerian degeneration and neuronal growth activation initiate repair [29]; phagocytic clearance of debris coincides with a transient oxidative burst [30]. Dedifferentiated Schwann cells align into contact guidance columns [31], and macrophages transition to a pro-regenerative M2 phenotype, marking a decisive microenvironmental switch [32,33]. Concurrent angiogenesis sustains axonal elongation [34]; late repair consolidates through remyelination [35]. Successful regeneration thus requires precise resolution of inflammatory and redox signals; their persistence entrains multicellular asynchrony, fibrosis, and irreversible functional deficit [36].

DPN originates from sustained hyperglycemic stress that reprograms the endoneurial microenvironment toward oxidative overload and smoldering inflammation [27]. Macrophages sustain M1-polarized cytokine secretion, amplifying axonal injury [37]; metabolic dysregulation drives mitochondrial reactive oxygen species (ROS) excess. Endothelial oxidative injury disrupts blood-nerve barrier integrity, imposing chronic hypoperfusion [38]. These pressures culminate in Schwann cell and neuronal mitochondrial dysfunction, degrading myelin, interrupting axonal transport, and attenuating conduction [39].

CIPN phenocopies DPN's immuno-redox circuitry but originates from acute mitochondrial insult by neurotoxic chemotherapeutics [40]. Direct oxidative phosphorylation inhibition triggers a sustained ROS surge, licensing innate immune signaling with robust M1-skewed macrophage infiltration [41]. Persistent neurotoxic injury dismantles Schwann cell axonal support, driving distal-to-proximal axonopathy and irreversible deficits [40].

Neuropathic pain, the dominant clinical phenotype common to DPN and CIPN, is anchored in unabated inflammatory and redox signaling that lowers nociceptor depolarization thresholds. Macrophages serve as pivotal effectors via interleukin-1 beta (IL-1β), tumor necrosis factor-alpha (TNF-α), and ROS release; mast cells amplify mediator bioavailability [42,43]. At the channel level, these mediators heighten excitability through discrete modulation [44]: TNF-α-dependent voltage-gated sodium channel 1.7 (NaV1.7) gain-of-function [45], interleukin-6 (IL-6)-sensitized transient receptor potential ankyrin 1 (TRPA1) gating [46], and IL-1β-facilitated voltage-gated calcium channel 3.2 (CaV3.2) conductance [47].

#### 2.1.2 Modulatory role of circadian rhythms in PN pathogenesis

Circadian regulatory networks impose hierarchical temporal control over PN pathophenotypes, programming diurnal oscillations into both regenerative competence and pain processing. Following PNI, this chronobiological gating manifests as clock-driven fluctuations in axonal outgrowth, with regenerative capacity peaking during the nocturnal phase [6]. In non-traumatic PN, circadian imprinting is illustrated in the temporal organization of neuropathic pain. Nocturnal exacerbation in DPN reflects deficient rhythmic antioxidant buffering in sensory ganglia, enabling oxidative stress maxima and nociceptor sensitization to coalesce at night [28,48]. In CIPN, chemotherapeutic agents dismantle peripheral circadian coherence and clock gene oscillators; this disruption propagates amplified neuroinflammation and nocturnal pain escalation, establishing a robust 24-h chronovariability signature [7].

### 2.2 Treatment of PNI

The formulation of lesion-adapted reconstructive strategies contingent upon injury phenotype and gap dimension remains a perennially unresolved challenge in PNI. Nerve injuries without gap typically respond to conservative rehabilitation. Complete transection, by contrast, requires surgical reapproximation: direct coaptation for short defects and autologous nerve grafting for substantive gaps [9,49]. Autograft repair, despite its gold-standard status, is constrained by donor site morbidity and limited tissue availability, factors that directly curtail functional restoration [50,51]. These inherent limitations have driven a technological shift toward alternative bridging platforms. Decellularized nerve allografts aim to preserve native guidance cues while mitigating immunogenicity; parallel efforts have yielded engineered nerve conduits that are graft-sparing, clinically integrated, and designed to support directed axonal outgrowth across defined gaps without autograft harvest [52].

**Fig. 2:**
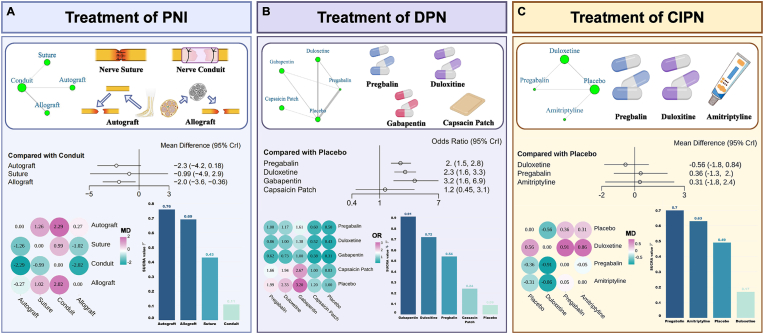
Network Meta-Analysis of Therapeutic Interventions for PNI, DPN, and CIPN. A. Surgical and scaffold-based interventions for PNI, including direct suture, autografts, allografts, and nerve conduits. B. Pharmacological treatments for DPN, including pregabalin, duloxetine, gabapentin, and capsaicin patches. C. Pharmacological agents assessed for CIPN and their comparative performance versus placebo. Across panels, forest plots depict effect estimates (Mean Differences (MD) for continuous outcomes or Odds Ratios (OR) for dichotomous outcomes) with credible intervals, while Surface Under the Cumulative Ranking Curve (SUCRA) rankings indicate the probability of each intervention achieving superior clinical efficacy. Created in https://BioRender.com.

To establish a quantitative evidence hierarchy among these modalities, a network meta-analysis was performed on 29 eligible studies (Fig. 2A) [9,49,[52], [53], [54], [55], [56], [57], [58], [59], [60], [61], [62], [63], [64], [65], [66], [67], [68], [69], [70], [71], [72], [73], [74], [75], [76], [77], [78]], with detailed search methods, eligibility criteria, and model diagnostics provided in the Supplementary Information (Fig. S1–S4). Sensory recovery, measured by static two-point discrimination, was compared across four interventions: autograft, direct suture, synthetic conduit, and decellularized allograft. Bayesian random-effects modeling revealed that nerve conduits yielded significantly inferior sensory recovery relative to decellularized allografts and showed no significant advantage over autografts or direct suture. Surface under the cumulative ranking curve (SUCRA) rankings consistently positioned autografts as the most effective and nerve conduits as the least effective for sensory reinnervation.

Critically, although current conduits restore macroscopic nerve continuity, they execute a passive biological mandate. Their capacity to counteract the hostile inflammatory-oxidative microenvironment that dictates regenerative success remains negligible [79]. This evidence-grounded performance gap crystallizes a distinct, addressable engineering challenge: the design of next-generation bioactive conduits capable of active, spatiotemporally regulated microenvironmental intervention.

### 2.3 Treatment of DPN

The therapeutic landscape for DPN remains exclusively symptomatic, with pharmacologic analgesia as the sole established modality [8]. First-line oral agents and topical capsaicin formulations constitute the standard of care [[80], [81], [82]]. A network meta-analysis of 22 trials confirmed that oral medications significantly outperform placebo and rank as the most effective options by SUCRA (Fig. 2B); capsaicin patches, however, fail to separate from placebo [[80], [81], [82], [83], [84], [85], [86], [87], [88], [89], [90], [91], [92], [93], [94], [95], [96], [97], [98], [99], [100], [101]]. Despite confirmed efficacy, these agents are compromised by adverse effects and a purely suppressive mechanism that alleviates pain without altering disease trajectory [8,80]. A fundamental therapeutic disconnect thus persists: transient pain relief remains decoupled from unremitting DPN progression. These compounds exert negligible impact on core pathogenic drivers, including chronic neuroinflammation, oxidative stress, circadian disruption, and progressive nerve fiber loss. This evidence-grounded void redirects innovation toward bioengineered interventions purpose-built for disease modification, rationally designed to redress the pathological endoneurial microenvironment through sustained, spatiotemporally regulated delivery of pleiotropic regenerative agents.

### 2.4 Treatment of CIPN

The complete absence of disease-modifying therapies renders CIPN a definitive translational engineering mandate [102]. Current clinical guidance offers marginal recourse, confined to chemotherapy attenuation and limited duloxetine recommendation [103]. To establish a quantitative efficacy benchmark, a network meta-analysis was performed. Pronounced heterogeneity mandated rigorous eligibility criteria, constraining the evidence base to five controlled studies (Fig. 2C) [[103], [104], [105], [106], [107]]. Comparative pain intensity analysis yielded no statistically robust evidence that duloxetine, pregabalin, or amitriptyline outperforms placebo; SUCRA rankings disclosed no efficacy hierarchy.

Notably, several factors warrant cautious interpretation of these findings. The analysis encompassed only five controlled studies, each constrained by limited sample sizes. As a result, the absence of statistically significant differences cannot be interpreted as evidence of comparable efficacy. Instead, it underscores the still low certainty and methodological constraints inherent to the current evidence base. Beyond these evidentiary limitations, the therapeutic landscape for CIPN remains currently narrow. Available pharmacological options are predominantly symptom-oriented and possess minimal capacity to meaningfully modify the neuroinflammatory, oxidative, and degenerative processes that sustain persistent nerve dysfunction.

Collectively, current therapeutic strategies for PN face three interconnected constraints: limited disease-modifying capacity, insufficient control over the lesional microenvironment, and poor adaptability to the divergent pathoanatomical demands of traumatic versus non-traumatic disorders. These limitations reveal a still-persistent unmet need for interventions that effectively reprogram the degenerative neural niche, moving beyond symptomatic palliation or passive structural support. Within this therapeutic impasse, melatonin emerges as a uniquely attractive candidate. Its appeal stems from coordinated regulation of inflammation, oxidative stress, circadian dysregulation, and multicellular repair mechanisms. Yet without delivery systems capable of confining bioavailability to the lesion, programming release kinetics, and imposing precise spatiotemporal control, melatonin's full therapeutic potential is unlikely to be realized. This convergence of biological rationale and engineering demand establishes the foundation for bioengineered melatonin platforms and increasingly motivates the bibliometric analysis presented here, which traces the field's evolution toward this emerging paradigm.

## 3 Comprehensive bibliometric analysis of melatonin in PN

This study performed a bibliometric analysis to map the research landscape of melatonin in PN and to trace its evolution from mechanistic inquiry toward delivery-oriented, biomaterial-enabled translational design. The Web of Science Core Collection (WoSCC) was interrogated across the 2001–2026 interval using a comprehensively assembled keyword lexicon encompassing melatonin, PN subtypes, and tissue engineering paradigms. After duplicate elimination, 571 publications were retained and computationally parsed using CiteSpace and Tableau for high-resolution visualization. For methodological transparency, a detailed protocol for the bibliometric landscape analysis is provided in the Supplementary Information (Fig. S5).

**Fig. 3:**
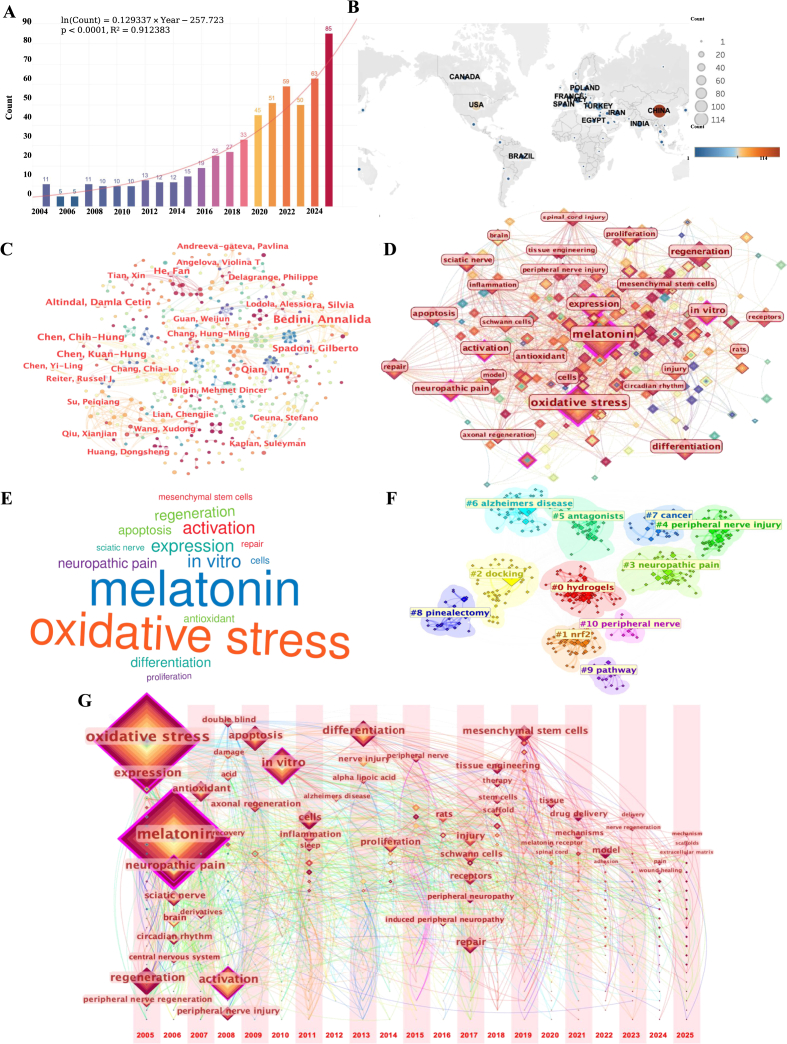
Bibliometric landscape of melatonin-related research in peripheral neuropathy. A. Annual publication output from 2004 to 2025. B. Global distribution of publications by country/region. C. Author co-authorship network showing collaborative clusters among high-output authors.D. Keyword co-occurrence network showing the conceptual structure of the research landscape. E. Word cloud of high-frequency keywords. F. Keyword clustering map identifying 11 thematic domains. G. Timeline visualization of keyword evolution.

Annual publication output exhibited a consistently ascending trajectory, with model-based forecasting supporting an exponential growth regime (Fig. 3A). China and the United States constituted the most productive geographic contributors, while co-authorship network decomposition revealed a polycentric collaborative architecture partitioned into multiple densely interconnected research communities (Fig. 3B and C). Keyword co-occurrence network topography positioned melatonin and oxidative stress as dominant conceptual nuclei, frequently co-registered with neuropathic pain, axonal repair, and sciatic nerve injury models (Fig. 3D, E, Fig. S6A and B). Cluster analysis yielded 11 thematic domains, with hydrogels representing the largest cluster. This result underscores the ascendance of biomaterial-enabled and delivery-oriented research directions in the field (Fig. 3F). Temporal evolution mapping, operationalized through timeline visualization, illuminated a decisive epistemic transition: progressive migration from foundational mechanistic inquiry toward convergent integration with tissue-engineered, spatiotemporally programmable delivery platforms (Fig. 3G, Fig. S6C and D).

The bibliometric insights substantiate an accelerating convergence between melatonin biology and biomaterials engineering within the PN research landscape. The findings reveal two interconnected trends. The first is an epistemic shift away from the mechanistic investigation of melatonin itself. The second is a translational shift toward the development of platforms capable of localized retention, programmable release, and microenvironment-directed intervention. This evolving research trajectory provides the conceptual basis for the present review. It prompts a focused examination of how material selection, delivery architecture, and release programming may be strategically integrated within melatonin-incorporated tissue engineering paradigms to engage the multifactorial and dynamically interconnected pathobiology of PN.

## 4 Engineered melatonin-loaded scaffolds for PNI

Aiming to engineer a rational roadmap for advanced melatonin delivery, this section establishes operationally defined concentration windows derived from quantitative cell-type pharmacodynamic profiling, anchoring subsequent scaffold design in mechanistically validated biological parameters. The contemporary melatonin-loaded scaffold (MLS) landscape for PNR is critically interrogated. To accelerate innovation, transferable architectural logic is systematically harvested from melatonin-functionalized platforms in non-neural tissue engineering contexts, enabling cross-domain synthesis of material, structural, and release-kinetic principles. The section concludes by analytically projecting MLS evolutionary vectors, delineating emergent design trajectories, and articulating an integrated, forward-looking engineering agenda for the rational development of precision-programmed, peripheral-nerve-specific delivery platforms.

### 4.1 Molecular targets and mechanistic actions of melatonin in PNI

Optimizing melatonin for PNR imposes rigorous engineering control over two codependent delivery variables: the spatial contour of local concentration and the temporal waveform of its presentation. Therapeutic activity is constrained within a cell-specific concentration envelope; circadian phase gates melatonin's biological output. A translatable intervention strategy thus demands a delivery architecture capable of enforcing sustained, lesion-compartmentalized bioavailability while synchronizing release with unfolding regenerative programs. Translating this mechanistic specification into quantifiable material design parameters is non-negotiable, requiring scaffold systems engineered to encode these spatiotemporal and dose-exposure mandates within their physical and chemical programming.

#### 4.1.1 Biosynthesis and sources of melatonin

Melatonin biosynthesis in mammals operates through a dual-source architectural logic, integrating central endocrine production with spatially distributed peripheral synthetic capacity. The canonical pineal cascade positions arylalkylamine N-acetyltransferase (AANAT) as the circadian-gated, rate-determining enzymatic node [108]; this axis, phase-locked to suprachiasmatic oscillators, drives the nocturnal surge in systemic melatonin [109]. Operating independently, melatonin synthesis or localized accumulation occurs across peripheral tissues [110]. In peripheral nerve, injury stress upregulates AANAT in presynaptic axons, indicating inducible competence for de novo melatonin generation within the compromised neural microenvironment [111]. Although the complete enzymatic itinerary in nerve elements awaits definitive mapping, the established capacity for mitochondrial melatonin synthesis in neurons confers biological plausibility upon injury-elicited, compartmentalized melatonin production in the peripheral nervous system [112].

#### 4.1.2 Endogenous melatonin in PNI pathogenesis

A defining operational feature of melatonin in PNR is its precise temporal synergy with the circadian architecture of endogenous regeneration. Following PNI, the physiological melatonin surge during the dark phase aligns with the chronobiologically privileged window of maximal axonal outgrowth [6,113]. Regenerative competence is thus gated by circadian phase and selectively amplified within this temporal niche. Therapeutic efficacy is therefore contingent not on concentration alone but critically on administration timing. Dark-phase delivery markedly accelerates functional recovery relative to daytime intervention, providing direct evidence for a circadian-restricted mechanism [114]. This chronologically encoded efficacy profile distinguishes melatonin from conventional neuroprotective agents and establishes its unique designation as a chronotherapeutically programmable agent for engineered PNR platforms [115]. Moving beyond the goal of ensuring adequate local melatonin exposure, future MLS design should prioritize the incorporation of temporally defined release strategies. Such strategies aim to synchronize melatonin delivery with biologically favorable circadian windows.

#### 4.1.3 Dose-graded cell-specific regulation governed by melatonin

**Fig. 4:**
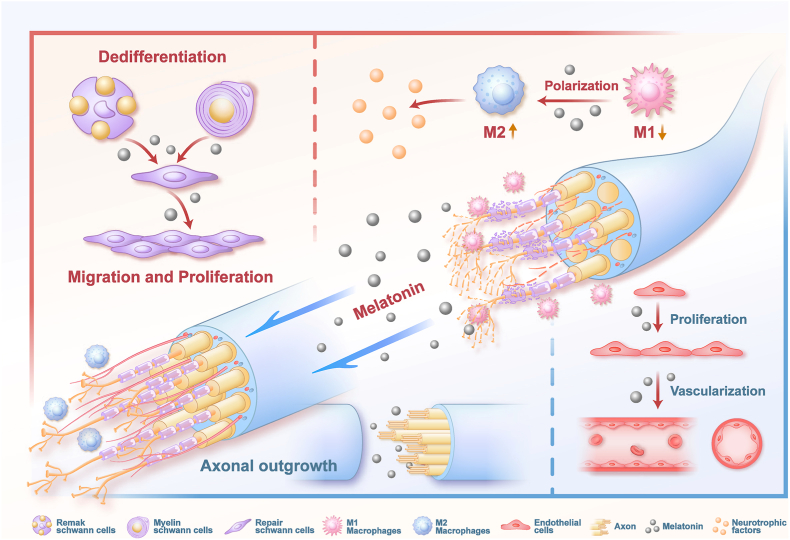
Schematic overview of the dose-dependent and cell-specific actions of melatonin on repair-relevant cell populations during peripheral nerve repair (PNR). In Schwann cells, melatonin supports dedifferentiation and the subsequent migration and proliferation programs required for axonal guidance and remyelination. In macrophages, melatonin promotes an M2-like reparative polarization and alters the expression of key cytokines and neurotrophic factors. In endothelial cells, melatonin promotes proliferative and pro-angiogenic responses that facilitate vascular remodeling and support axonal outgrowth.

A quantitatively resolved pharmacodynamic map of melatonin's dose-dependent effects across principal neural cell lineages constitutes an essential design specification for precision delivery platforms. These concentration-graded response profiles delineate operational therapeutic windows that constrain release kinetic programming. Available in vitro data from macrophages, Schwann cells, endothelial cells, and neurons were systematically aggregated, yielding experimentally anchored exposure corridors for biomaterial scaffold design (Fig. 4).

Following PNI, melatonin exerts tiered, concentration-dependent modulation of macrophage polarization. Lower concentrations (0.01–1 μM) restrain M1 activation and cytokine output [13]; moderate concentrations (10–150 μM) redirect toward M2-skewed repair phenotypes while sustaining M1 suppression, representing the most referenced window for pro-regenerative immunomodulation [[116], [117], [118]]; higher concentrations (0.2–1.0 mM) elicit broad-spectrum immunosuppressive effects [[119], [120], [121]].

Schwann cells, as principal PNR executors, exhibit concentration-stratified responses. Minimal concentrations (1–10 nM) augment proliferation and survival [122]; low micromolar levels (5–10 μM) redirect toward repair-specialized phenotypes with enhanced migration and cytoprotection, potentiating myelin preservation and remyelination [[123], [124], [125]].

Endothelial cells, essential architects of the vascular niche, respond to melatonin as a titratable programmer. Low concentrations (0.01–0.03 μM) stabilize endothelium through enhanced survival and barrier function [126,127]; moderate concentrations (1–10 μM) promote proliferation and migration to sustain angiogenesis [128,129]; higher concentrations (60–100 μM) amplify angiogenic capacity through combinatorial antioxidative and anti-inflammatory action [[130], [131], [132]].

Neuronal survival and axonal integrity are prerequisites for functional recovery. Melatonin provides dose-graded neuroprotection and pro-regenerative activity. In vitro, 10 μM potentiates neuronal proliferation and differentiation [133]; in vivo, 1 mg/kg/day is associated with pro-regenerative action [134], whereas higher doses (50–100 mg/kg/day) bias toward cytoprotection [135,136].

**Fig. 5:**
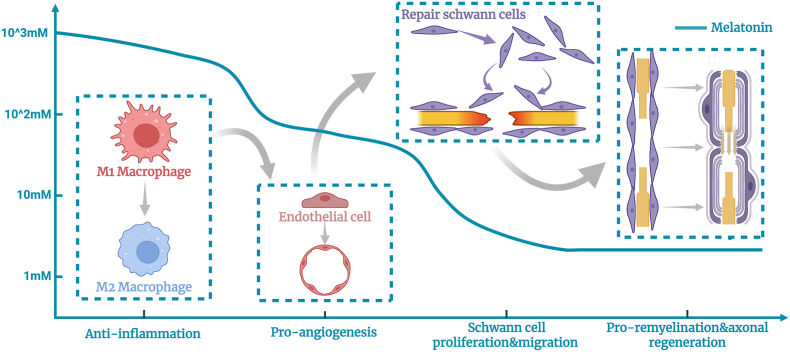
Stage-specific alignment of melatonin release kinetics with peripheral nerve regeneration. Created in https://BioRender.com.

Dose-correlated in vitro signaling networks are catalogued in Table 1, Table 2; in vivo outcomes are in Table 3. Cross-cellular synthesis exposes a foundational engineering imperative: therapeutic concentration must exhibit phase-specific adaptation [137]. Acute intervention mandates high local concentrations (100 μM–1 mM) to abort nascent inflammatory-oxidative cascades; the protracted regenerative phase requires a deliberate downshift to sustained moderate levels (1–10 μM) to potentiate Schwann cell, endothelial, and neuronal engagement. This temporal specification is incompatible with static release profiles. Clinically translatable platforms must therefore be engineered for adaptive dosing, affording precise spatiotemporal control to achieve phase-locked alignment with sequential regenerative demands. To clarify this stage-dependent dosing principle, a schematic concentration-time curve is introduced. The curve maps melatonin release kinetics onto the sequential phases of nerve regeneration, featuring an early high-exposure phase to suppress inflammatory and oxidative injury, followed by sustained moderate-to-low exposure to support subsequent regenerative events (Fig. 5).

**Table 1:** In vitro dose-dependent signaling pathways of melatonin in macrophages and Schwann cells.

Cell line		Dosage	Signal Path	Ref
Macrophages	RAW264.7	0.01-1 μM	Melatonin activates SIRT1 and suppresses Notch signaling, thereby limiting M1 polarization.	[13]
	Human monocyte- derived macrophages	10 μM	Melatonin activates RORα and AMPKα, inhibits STAT1 while promoting STAT3, shifting macrophages away from M1 toward M2.	[116]
	Thioglycollate-induced mouse peritoneal macrophages	100 μM	Melatonin inhibits NF-κB/p38 MAPK and activates Nrf2/HO-1, reducing pro-inflammatory mediators and enhancing antioxidant defense, ultimately restraining M1 programming.	[117]
	RAW264.7	100 μM,150 μM	Melatonin suppresses Src phosphorylation and promotes FUNDC1-dependent mitophagy, lowering mitochondrial ROS and favoring M2 polarization.	[118]
	RAW264.7	200 μM	Melatonin downregulates ERK5 signaling, selectively enhancing IL-10 secretion in M2 macrophages.	[120]
	RAW264.7	1000 μM	Melatonin inhibits NF-κB (p50 activity) and STAT1 phosphorylation, downregulating iNOS and IL-6 expression.	[121]
Schwann cell	RSC96	1 nM	Melatonin signals via MT1 to activate ERK1/2, promoting Schwann cell proliferation.	[122]
	RT4-D6P2T	0.5-10 μM	Melatonin acts through MT1/MT2 to engage Ras/Raf/ERK and GDNF/PKC signaling, supporting dedifferentiation and proliferation.	[123]
	RT4-D6P2T	10 μM	Melatonin activates NF-κB and FAK signaling, upregulating SOX2 and driving a proliferative, dedifferentiated phenotype.	[124]
	RSC96	2.5-20 μM	Melatonin upregulates Parkin to enhance mitophagy, reduces mitochondrial ROS, and supports survival and myelin regeneration.	[125]

**Abbreviations:** SIRT1: sirtuin 1; RORα: retinoic acid receptor-related orphan receptor alpha; AMPKα: AMP-activated protein kinase alpha; STAT1: signal transducer and activator of transcription 1; STAT3: signal transducer and activator of transcription 3; NF-κB: nuclear factor kappa B; Nrf2: nuclear factor erythroid 2-related factor 2; HO-1: heme oxygenase-1; FUNDC1: FUN14 domain-containing 1; ROS: reactive oxygen species; ERK5: extracellular signal-regulated kinase 5; IL-10: interleukin-10; iNOS: inducible nitric oxide synthase; IL-6: interleukin-6; MT1: melatonin receptor 1; MT2: melatonin receptor 2; ERK1/2: extracellular signal-regulated kinase 1/2; Ras: rat sarcoma; Raf: rapidly accelerated fibrosarcoma; GDNF: glial cell line-derived neurotrophic factor; PKC: protein kinase C; FAK: focal adhesion kinase; SOX2: SRY-box transcription factor 2; Parkin: Parkinson juvenile disease protein 2.

**Table 2:** In vitro dose-dependent signaling pathways of melatonin in endothelial cells and neurons.

Cell line		Dosage	Signal Path	Ref
Endothelial cell	Rat mesenteric endothelial cells	30 nM	Melatonin acts via MT1/MT2 to modulate intracellular Ca^2+^, inhibiting P2Y1-dependent leukocyte adhesion and TNF-α release.	[127]
	CMEC	1-10 μM	Melatonin activates MAPK/ERK and suppresses CREB-dependent Ca^2+^-overload signaling (IP3R/VDAC), preserving mitochondrial function and limiting apoptosis.	[128]
	GEC	10 μM	Melatonin upregulates MT1/MT2, improves mitochondrial membrane potential, and activates VEGF–survivin signaling to promote angiogenesis.	[129]
	HUVECs	100 μM	Melatonin activates Nrf2, enhances antioxidant enzymes, reduces ROS, and suppresses NLRP3 inflammasome–driven.	[130]
	HUVECs	60 μM	Melatonin activates SIRT1/SIRT3, dampening oxidative stress/inflammation/apoptosis while protecting mitochondrial function and supporting angiogenic competence.	[131]
	hCMEC/D3	100 μM	Melatonin upregulates BMP6 and activates Smad1/5/9 signaling, increasing VEGF/Ang1 expression and enhancing endothelial migration.	[132]
Neuron	PC12	10 μM	Melatonin signals via MT receptors to activate MEK/ERK and PI3K/AKT, supporting proliferation; preferential MEK/ERK activation also promotes MAP2 expression and neurite outgrowth.	[133]

**Abbreviations:** CMEC: cardiac microvascular endothelial cells; GEC: glomerular endothelial cells; HUVECs: human umbilical vein endothelial cells; hCMEC/D3: human cerebral microvascular endothelial cell line D3; PC12: pheochromocytoma 12 cells; nM: nanomolar; μM: micromolar; MT: melatonin receptor; MT1: melatonin receptor 1; MT2: melatonin receptor 2; Ca^2+^: calcium ion; P2Y1: purinergic receptor P2Y1; TNF-α: tumor necrosis factor-alpha; MAPK: mitogen-activated protein kinase; ERK: extracellular signal-regulated kinase; CREB: cAMP response element-binding protein; IP3R: inositol 1,4,5-trisphosphate receptor; VDAC: voltage-dependent anion channel; VEGF: vascular endothelial growth factor; Nrf2: nuclear factor erythroid 2-related factor 2; ROS: reactive oxygen species; NLRP3: NLR family pyrin domain containing 3; SIRT1: sirtuin 1; SIRT3: sirtuin 3; BMP6: bone morphogenetic protein 6; Smad1/5/9: SMAD family member 1/5/9; Ang1: angiopoietin-1; MEK: mitogen-activated protein kinase kinase; PI3K: phosphoinositide 3-kinase; AKT: protein kinase B; MAP2: microtubule-associated protein 2.

**Table 3:** In Vivo Dose-Dependent Biological effect of Melatonin Across Peripheral Nerve-Relevant Cell Types.

Cell	Animal species	Dosage	Biological effect	Ref
Macrophages	Sprague-Dawley	10 mg/kg/day (intraperitoneal injection)	Inhibit M1 macrophage polarization, promote M2 macrophage polarization	[13]
	ApoE^−/−^ mice, RORα^sg/sg-ApoE^−/−^ mice	10 mg/kg/day (intragastric administration)	Inhibit M1 macrophage polarization, promote M2 macrophage polarization; Upregulate RORα expression in macrophages	[116]
	DBA/1	15,30 mg/kg/day (intraperitoneal injection)	Inhibit M1 macrophage polarization, promote M2 macrophage polarization; Upregulate the Fundc1 expression in macrophages.	[118]
	Sprague-Dawley	2.5,10 mM, 200 μL once every two days (local injection)	No effect on M2 macrophage polarization; promotes IL-10 release by M2 macrophages.	[120]
Schwann cells	Wistar	1,10 mg/kg/day (intraperitoneal injection)	Significantly enhances Schwann cell proliferation; improves nerve repair efficacy; increases the number of innervated motor end plates.	[122]
	Sprague-Dawley	10 mg/kg/day (intraperitoneal injection)	Upregulating Parkin expression activates mitophagy; inhibits mitochondrial apoptosis; promotes myelin regeneration; enhances neurological function recovery.	[125]
Endothelial cell	C57BL/6	20 mg/kg/day (intraperitoneal injection)	Upregulating the SIRT1/SIRT3 signaling pathway, promoting angiogenesis in the ischemic region, and reducing cell apoptosis and inflammation	[131]
	C57BL/6	20,40 mg/kg/day (intraperitoneal injection)	Promotes endothelial cell proliferation, increases microvascular diameter, and upregulates BMP6, Smad1/5/9, VEGF, and Ang1.	[132]
Neuron	Wistar	1 mg/kg/day (intraperitoneal injection)	Through the MT1 receptor-dependent pathway, it inhibits CaMKII activation, upregulates the expression of β3-tubulin and GAP43, accelerates axon sprouting and cytoskeletal remodeling, and promotes motor end plate reinnervation.	[134]
	Wistar	50 mg/kg/day (intraperitoneal injection)	Reduce the lipid peroxidation product, enhance the activity of antioxidant enzymes; alleviate oxidative stress-induced damage in nerve tissue, and promote repair after nerve injury.	[135]
	Wistar	100 mg/kg/day (intraperitoneal injection)	Inhibits nNOS activation, preserves the antioxidant activity of SOD, maintains ChAT expression, alleviates oxidative stress-induced damage, and accelerates nerve-muscle reinnervation.	[136]

**Abbreviations:** ApoE−/−: apolipoprotein E knockout; RORα: retinoic acid receptor-related orphan receptor alpha; sg/sg: staggerer homozygous mutation genotype; RORαsg/sg-ApoE−/− mice: retinoic acid receptor-related orphan receptor alpha staggerer homozygous/apolipoprotein E knockout mice; mg/kg/day: milligrams per kilogram per day; mM: millimolar; μL: microliter; IL-10: interleukin-10; FUNDC1: FUN14 domain containing 1; Parkin: Parkinson juvenile disease protein 2; SIRT1: sirtuin 1; SIRT3: sirtuin 3; BMP6: bone morphogenetic protein 6; Smad1/5/9: SMAD family member 1/5/9; VEGF: vascular endothelial growth factor; Ang1: angiopoietin-1; MT1: melatonin receptor 1; CaMKII: calcium/calmodulin-dependent protein kinase II; β3-tubulin: beta-3 tubulin; GAP43: growth associated protein 43; nNOS: neuronal nitric oxide synthase; SOD: superoxide dismutase; ChAT: choline acetyltransferase.

### 4.2 Advanced nerve scaffold design specifications

In tissue engineering, scaffolds function as localizable, long-acting delivery platforms for the sustained presentation and kinetically programmed release of bioactive cues throughout the regenerative continuum. Melatonin's therapeutic efficacy is constrained by two interdependent operational variables: local lesion concentration and circadian phasing. Scaffold-based delivery enforces durable lesion-restricted retention and enables temporally adaptive release aligned with neural repair milestones. Rational design must therefore converge controlled-release engineering with mechanobiological determinants of successful PNR [138].

**Fig. 6:**
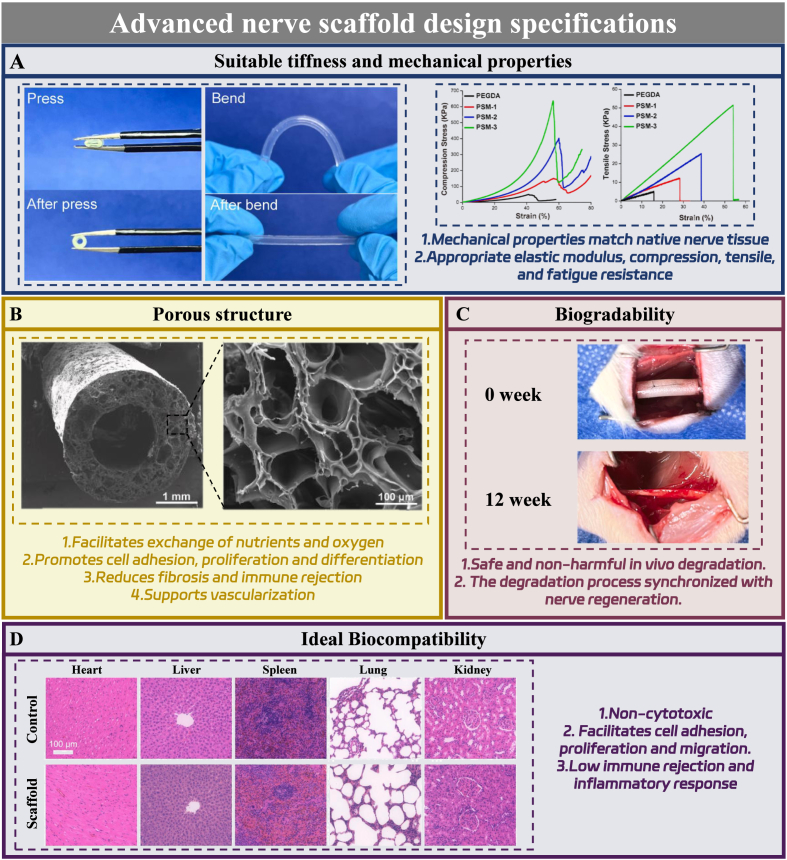
Design principles for tissue-engineered scaffolds supporting PNR. A. Mechanical properties compatible with native peripheral nerve tissue. Reproduced with permission from Ref. [138]. B. An interconnected porous architecture that facilitates oxygen and nutrient transport. Reproduced with permission from Ref. [138]. C. Controlled biodegradation synchronized. Reproduced with permission from Ref. [138]. D. Long-term biocompatibility. Reproduced with permission from Ref. [139].

Mechanical specification constitutes a primary design tier. Scaffolds must replicate native neural tissue compliance and resilience while withstanding physiological loads [138]. These properties prevent secondary axonal compression and avoid stress shielding that impedes adaptive remodeling. Preservation of mechanical fidelity throughout the early-to-intermediate repair phase ensures uninterrupted axonal guidance and robust host integration (Fig. 6A).

Pore architecture defines a second design tier. An interconnected porous network sustains oxygen and nutrient perfusion while providing a permissive substrate for Schwann cell adhesion, migration, and expansion [138]. This architecture mitigates fibrotic encapsulation and maladaptive immune recognition while allocating space for vascular ingrowth. In melatonin-functionalized scaffolds, pore geometry directly programs intraluminal drug distribution and release kinetics, governing both spatial uniformity and temporal endurance of local melatonin exposure (Fig. 6B).

Degradation kinetics constitute a third design tier. Precise temporal alignment of scaffold resorption with the endogenous regenerative timeline is mandatory [138]. Premature degradation compromises axonal bridging; excessive persistence obstructs matrix remodeling and perpetuates foreign-body reactions. In melatonin-integrated systems, degradation behavior is coupled to drug elution dynamics. A calibrated degradation profile sustains therapeutically adequate local melatonin concentration across the full reparative sequence, from acute inflammatory-oxidative modulation to axonal regeneration and remyelination (Fig. 6C).

Biocompatibility represents a fourth, cross-cutting design tier. Implantable neural constructs must exhibit negligible cytotoxicity while actively supporting neural cell adhesion and guided migration, with limited foreign-body encapsulation [139]. This requirement assumes heightened criticality in melatonin-eluting systems, where unrestrained inflammation or progressive fibrotic sequestration could simultaneously undermine structural integration and neutralize local immunomodulatory and antioxidative actions (Fig. 6D).

### 4.3 Therapeutic applications of MLS for PNI

The functional specification of nerve scaffolds has decisively advanced from passive physical bridging toward active, multidimensional reprogramming of the regenerative microenvironment. Melatonin, operating as a pleiotropic biological instruction set within this paradigm, delivers integrated anti-inflammatory and antioxidative signaling that synergistically potentiates both histological and functional recovery. To systematically deconstruct current design architectures, MLS strategies for PNI are categorized into three compositionally and functionally distinct paradigms: polymeric scaffolds, physically augmented scaffolds, and cell-integrated composite systems. This tripartite framework captures a progressive escalation in the integrative coupling of topographical guidance with microenvironmental engineering.

From a biofabrication perspective, electrospinning constitutes the predominant manufacturing strategy in current melatonin-based nerve scaffold design. This prominence derives from its capacity to generate extracellular matrix (ECM)-mimetic fibrous matrices with tunable alignment, porosity, and drug-loading characteristics [140,141]. Such fibrous architectures recapitulate key structural features of the native neural extracellular milieu, thereby providing topographical guidance for directional axonal growth while simultaneously functioning as reservoirs for localized and sustained melatonin release [20,142]. In comparison, three-dimensional (3D) printing appears less frequently in the present body of MLS studies. Nevertheless, it offers complementary advantages in geometric customization, multichannel conduit fabrication, and spatially defined integration of biological components [143]. Fabrication methodologies and corresponding biological effect profiles across these scaffold classes are comprehensively compiled and synthesized in Table 4.

**Table 4:** Representative MLS designs for PNI.

Scaffold	Materials composition	Fabrication method	Biological effects	Ref
MLT/PCL	PCL	Multilayer molding	Promotes Schwann cell viability and proliferation; attenuates oxidative stress and inflammation; improves mitochondrial function; activates autophagy and suppresses apoptosis; improves functional/electrophysiological recovery.	[144]
PGMLT	PCL,Gelatin	Electrospinning	Promotes Schwann cell viability and proliferation; enhances neural differentiation and neurite outgrowth.	[20]
MAH/PCL	PCL, Alginate hydrogel	Outlayer: Electrospinning(PCL) Inner layer: Crosslinked by CaCl_2_	Controlled melatonin release (∼80% released within 15 days); promotes axonal regeneration and remyelination; restores inflammatory–oxidative microenvironment balance; enhances vascularization.	[14]
ML-NGC	PCL, Fe3O4-MNPs	Electrospinning	Promotes cell proliferation; enhances nerve regeneration and remyelination; mitigates inflammatory and oxidative stress via pro-repair immune polarization.	[145]
MLT/rGO/PCL	PCL, rGO	Electrospinning	Improves mitochondrial membrane potential and reduces mitochondrial depolarization; inhibits apoptosis and supports cellular proliferation; enhances axonal outgrowth and myelination; reduces inflammatory burden.	[21]
rGO/PCL/Mel	PCL, rGO	3D coaxial printing	Promotes Schwann cell adhesion and proliferation on the scaffold; Early burst melatonin release(∼69% in 24 h); improves functional and electrophysiological recovery.	[143]
L-ZBZPGM	L-zein, Zein/Pectin, GO	Electrospinning	Promotes Schwann cell viability and proliferation; reduces oxidative stress and improves mitochondrial status; enhances anti-inflammatory performance; supports neurogenic differentiation-like morphology under electroactive cues.	[146]
PU/GNFs	Polyurethane, Gelatin nanofibrils, Schwann cells	Electrospinning	Promotes Schwann cell adhesion and pro-regenerative morphology; reduces oxidative stress and improves mitochondrial function; improves functional recovery; mitigates denervation-related muscle atrophy.	[147]
ADSCs/rhNGF@PMCS NFs	PCL, Chitosan, r-ADSCs	Electrospinning	Supports Schwann cell viability, proliferation and migration; improves functional recovery and sensory outcomes.	[148]

**Abbreviation:** MLT/PCL: melatonin/polycaprolactone nerve guide conduit; PGMLT: Melatonin-loaded gelatin-polycaprolactone electrospun fibrous scaffold; MAH/PCL: melatonin-loaded alginate hydrogel-based PCL nanofiber composite scaffold; ML-NGC: multilayered composite nerve guidance conduit loaded with melatonin and Fe3O4 magnetic nanoparticles; MLT/rGO/PCL: melatonin and reduced graphene oxide-loaded polycaprolactone nerve guide conduit; rGO/PCL/Mel: reduced graphene oxide/polycaprolactone/melatonin hybrid aerogel nerve fiber; L-ZBZPGM: hydrogel composite nerve conduit composed of cysteinylated zein, berberine, Zein, Pec, graphene oxide, and melatonin; PU/GNFs: polyurethane/gelatin nanofibril neural guidance conduit; ADSCs/rhNGF@PMCS NFs: rhNGF/melatonin-coated Chitosan/PCL nanofibrous nerve conduit loaded with rat adipose-derived stem cells.

#### 4.3.1 Polymeric MLS platforms

**Fig. 7:**
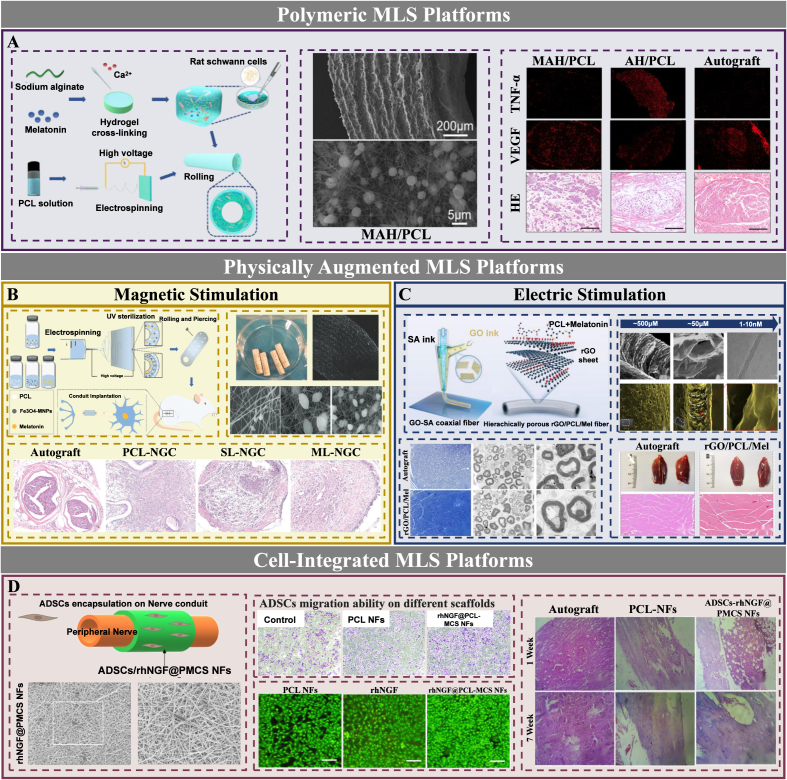
Design Strategies of Melatonin-loaded scaffolds (MLS) for PNR. A. Polymeric MLS to enable sustained local delivery and coordinate inflammatory suppression, oxidative stress control, and pro-angiogenic vascular remodeling, thereby supporting axonal regeneration. Reproduced with permission from Ref. [14]. MAH/PCL: melatonin-loaded alginate hydrogel-based PCL nanofiber composite scaffold. B. MLS integrated with magnetic stimulation, combining biochemical regulation with directional physical cues to enhance axonal alignment and functional recovery. Reproduced with permission from Ref. [145]. SL-NGC: single-layer nerve guidance conduit. ML-NGC: multi-layer nerve guidance conduit. C. Electrically conductive MLS that couples controlled drug release with bioelectrical stimulation to promote axonal maturation and remyelination. Reproduced with permission from Ref. [143]. rGO: reduced graphene oxide. D. Cell-integrated MLS that combines regenerative cell support with melatonin-mediated immuno-redox regulation to reinforce neurotrophic signaling and functional repair. Reproduced with permission from Ref. [148]. ADSCs/rhNGF@PMCS-NFs: adipose-derived stem cells/recombinant human nerve growth factor@PCL/melatonin/chitosan nanofibers.

Early MLS designs for PNI employed monolithic polycaprolactone (PCL) matrices to achieve sustained local delivery. Melatonin incorporation significantly improved functional recovery to levels comparable with autograft repair, mechanistically attributed to enhanced antioxidant capacity and attenuated inflammatory activation within the lesion microenvironment [144]. To address the inherent biological inertness of hydrophobic polymers, subsequent iterations introduced hydrophilic components. Gelatin-PCL composites exhibited improved surface wettability and porosity, thereby facilitating cellular adhesion, migration, and neurite outgrowth while sustaining drug release over operative timeframes [20]. The most recent generation has adopted hierarchical architectures that functionally decouple structural support from drug delivery. A bilayer paradigm (Fig. 7A), integrating a mechanically competent PCL sheath with a hydrogel core, enables biphasic release kinetics and achieves autograft-equivalent regenerative outcomes through coordinated microenvironmental reprogramming encompassing inflammation resolution, oxidative stress buffering, and microvascular remodeling [14].

#### 4.3.2 Physically augmented MLS platforms

The convergence of physical stimulation with melatonin delivery establishes a multimodal regenerative paradigm that integrates biophysical cueing with microenvironmental biochemical instruction. Magnetically responsive systems, including Fe_3_O_4_/melatonin/PCL conduit (Fig. 7B), enable field-guided axonal alignment and achieve autograft-equivalent functional recovery, with rapid drug elution supporting pro-healing macrophage repolarization [145]. Electrically conductive scaffolds pursue a parallel strategy designed to reconstitute native bioelectrical signaling.

Graphene-family nanomaterials, particularly reduced graphene oxide (rGO), have emerged as promising components in electroactive nerve repair. This promise derives from their combined electrical conductivity and biocompatibility [149]. Mechanistically, rGO-mediated electrical cueing in neurons has been linked to activation of the mitogen-activated protein kinase signaling pathway [150]. Graphene oxide quantum dots have been reported to promote Schwann cell proliferation through the Wnt signaling pathway [151]. They also stimulate macrophage-derived vascular endothelial growth factor release via the extracellular signal-regulated kinase/cAMP response element-binding protein/vascular endothelial growth factor axis, thereby supporting neurovascularization [152]. Additionally, rGO-containing scaffolds may regulate the fate of stem and progenitor cells involved in nerve repair, including embryonic stem cells, mesenchymal stem cells, adipose-derived stem cells, and neural stem cells [150,153].

Initial melatonin/rGO/PCL composites established the feasibility of coupling conductivity with sustained drug release [21]. Subsequent architectural refinement introduced three-dimensional coaxial printing as an advanced manufacturing strategy for PNR. The resulting rGO/PCL/melatonin hybrid aerogel-fiber scaffold integrated hierarchically ordered hollow channels, ultrahigh porosity, mechanical compliance, and localized melatonin elution. This design more effectively coupled structural guidance with microenvironmental modulation and achieved regenerative outcomes comparable to autografting in a long-gap defect model (Fig. 7C) [143]. Bio-derived conductive composites have recently emerged to effectively address synthetic material constraints [154]. These systems preserve melatonin's immuno-redox functionality under electrical stimulation while cooperatively enhancing guided axonogenesis [146].

#### 4.3.3 Cell-integrated MLS platforms

The convergence of cellular components with melatonin delivery establishes an integrated biohybrid scaffold paradigm wherein architectural support, microenvironmental programming, and cell-mediated repair are functionally convergent. The scaffold furnishes a three-dimensional niche permissive for cell retention and organization. Melatonin preconditioning attenuates inflammatory and oxidative stress, thereby biasing the local microenvironment and cellular phenotypes toward regenerative commitment. Schwann cell-based designs leverage melatonin's cytoprotective activity to sustain effector cell function, with complementary trophic signals supplied through ancillary bioactive incorporation [147]. Alternative strategies employ adipose-derived stem cells for their differentiation capacity. A representative system integrates multi-agent controlled release with stem cell delivery, wherein melatonin establishes a permissive microenvironment and directs lineage specification while concomitant neurotrophic factor exposure provides explicit axonal growth instruction, yielding coordinated multi-factor regenerative potentiation (Fig. 7D) [148].

### 4.4 MLS engineering strategies beyond neuropathy

The engineering sophistication of MLS systems for PNI remains substantially below the developmental threshold established in other regenerative fields. In spinal cord, bone, and muscle applications, MLS research has traversed a markedly broader design space, yielding diverse material formulations and release programming architectures purpose-built to optimize local bioavailability and stage-specific therapeutic engagement. This extant knowledge base constitutes a directly transferable engineering lexicon, furnishing strategic design blueprints that can be systematically redeployed to accelerate MLS platform evolution specifically for PNR. Representative engineering platforms are comparatively summarized in Table 5.

**Table 5:** Representative MLS across different tissue-engineering contexts.

Platform	Tissue/Organ	Pathological condition	Melatonin delivery mode	Ref
Lap/MS@Mel	Spinal cord	Spinal cord injury	microsphere-mediated delivery	[155]
Mel/Ibu@D/P-gPSB		spinal cord injury	Diffusion-controlled release	[156]
MEL/BMP-2-chitosan/HAp scaffolds	Bone	Bone defect	Diffusion-controlled release	[157]
MT@PLGA NPs-PCL/β-TCP/SA		Diabetic bone defect	microsphere-mediated delivery	[158]
Ti/Gel/ZM		Osteoporosis	pH-responsive release	[159]
GH-MCD hydrogel		Infectious bone defect	pH-responsive release	[160]
GelMA/Mel@M2-exos		Periodontitis	Extracellular vesicle-mediated delivery	[161]
PEG-pp@nMSC@MT		Maxillofacial bone defect	Inflammation-responsive release	[162]
SF-GelMA@MT	Cartilage	Cartilage injury	Diffusion-controlled release	[163]
HG@MT-sEV/PCLUW		Cartilage injury	Extracellular vesicle-mediated delivery	[164]
mPDA@Mel-AB NPs/OCS-CMC/BG		Osteoarthritis	pH-responsive release	[165]
MLT@JFM	Tendon	Tendon injury	Diffusion-controlled release	[166]
GelMA-Lipo@MT	Muscle	Volumetric muscle loss	Liposome-mediated delivery	[167]
PT-MLT		Myocardial infarction	ROS-responsive release	[168]

**Abbreviation:** Lap/MS@Mel: Laponite hydrogel incorporated with melatonin-loaded PLGA microspheres; Mel/Ibu@D/P-gPSB: Melatonin/Ibuprofen co-loaded Dex/Per-g-PSB dynamic hydrogel platform; MEL/BMP-2-chitosan/HAp scaffolds: Melatonin and Bone morphogenetic protein 2 loaded chitosan/hydroxyapatite scaffolds; MT@PLGA NPs-PCL/β-TCP/SA: Melatonin-loaded PLGA nanoparticles incorporated with sodium alginate hydrogel and contained within polycaprolactone/β-Tricalcium phosphate scaffold; Ti/Gel/ZM: Titanium/GelMA/ZIF-8-Melatonin scaffold; GH-MCD hydrogel: GelMA–oxidized hyaluronic acid–melatonin carbon dots composite hydrogel; GelMA/Mel@M2-exos: GelMA hydrogel loaded with M2 macrophage-derived exosomes and melatonin-loaded M2 exosomes; PEG-pp@nMSC@MT: inflammation-responsive hydrogel loaded with functional MSC-targeted nanovesicles; SF-GelMA@MT: Silk Fibroin-GelMA@Melatonin composite scaffold; HG@MT-sEV/PCLUW: HAMA/GelMA double network hydrogel loaded with MT-pretreated cell-derived small extracellular vesicles/Poly (ε-caprolactone)/hydroxyapatite ultralong nanowire composite scaffold; mPDA@Mel-AB NPs/OCS-CMC/BG: oxidized chondroitin sulfate-carboxymethyl chitosan, loaded with mesoporous polydopamine@melatonin-ammonia borane nanoparticles and bioactive glass; MLT@JFM: Melatonin-loaded Janus fibrous membrane; GelMA-Lipo@MT: Gelatin methacryloyl hydrogel with liposome-encapsulated melatonin; PT-MLT: polyvinyl alcohol-TSPBA-melatonin hydrogel.

#### 4.4.1 Spinal cord

**Fig. 8:**
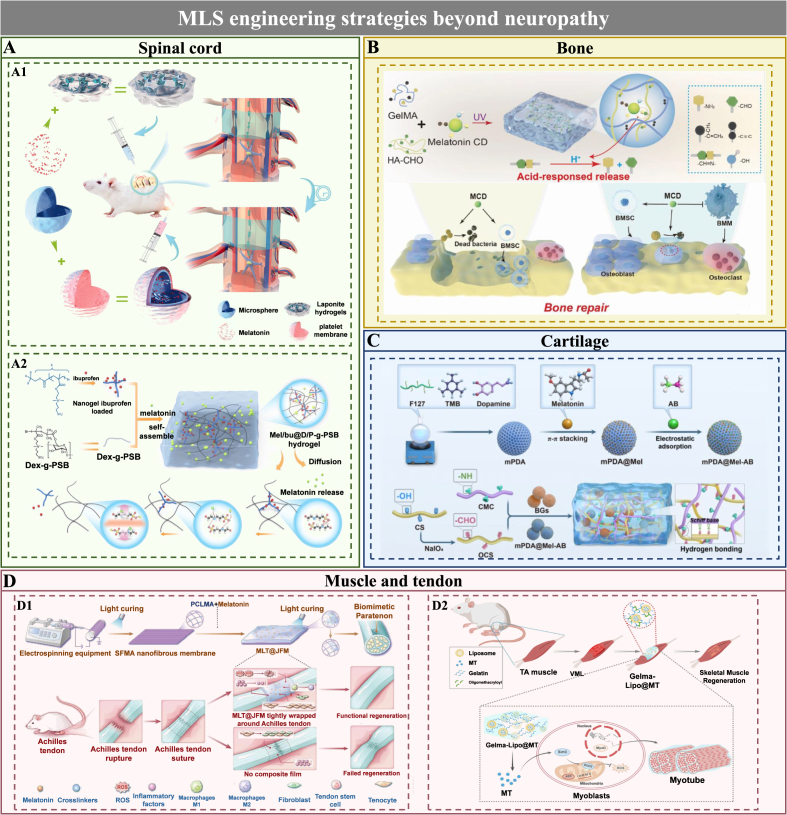
Schematic overview of MLS engineering strategies beyond neuropathy. A. MLS for spinal cord injury that enables localized, staged delivery to regulate secondary injury cascades and support neural repair. Reproduced with permission from Refs. [155,156]. B. MLS for bone regeneration, using responsive hydrogel or composite architectures to coordinate immuno-redox modulation and osteogenic remodeling in challenging microenvironments. Reproduced with permission from Ref. [160]. C. MLS for cartilage repair, designed to preserve chondrocyte homeostasis and enhance matrix regeneration through antioxidant, anti-inflammatory, and microenvironment-responsive delivery strategies. Reproduced with permission from Ref. [165] D. MLS for muscle and tendon repair, engineered to maintain soft-tissue mechanical compatibility while limiting inflammation and oxidative injury and promoting structural and functional restoration. Reproduced with permission from Refs. [166,167].

Following spinal cord injury, a hostile microenvironment defined by intense inflammation and oxidative stress propagates secondary neural injury [169,170]. PNI exhibits a convergent immuno-redox imbalance wherein the lesional niche critically dictates regenerative outcome [155,171]. In spinal cord injury, this pathological state manifests as sustained pro-inflammatory microglial activation with deficient reparative polarization, collectively impeding resolution [172,173]. Melatonin has accordingly emerged as a pleiotropic modulator capable of rebalancing these cascades and redirecting microglial phenotype commitment. Injectable hydrogels, offering localized retention and programmable release, have been extensively deployed as delivery platforms for spinal cord injury. One strategy encapsulated melatonin within polymer microspheres embedded in a nanocomposite hydrogel matrix, achieving enhanced lesion confinement and tunable release kinetics while enabling trans-barrier delivery [155] (Fig. 8A1). This construct attenuated inflammatory and oxidative injury and promoted both tissue preservation and functional recovery [155]. A more sophisticated design employed a chemically integrated zwitterionic hydrogel platform enabling dual functionality: mechanical compliance matching and electrostatic secondary drug loading. The resultant biphasic release profile delivered rapid initial melatonin elution to drive early microglial repolarization, followed by delayed secondary anti-inflammatory agent delivery upon progressive matrix degradation (Fig. 8A2). Through temporally coordinated immuno-redox reprogramming and sustained inflammatory suppression, this staged delivery architecture effectively mitigated secondary injury and enhanced motor recovery [156].

#### 4.4.2 Bone

The material and fabrication commonalities between bone and peripheral nerve scaffolds establish melatonin-enabled bone constructs as a transferable engineering paradigm for PNR delivery systems. In bone repair, melatonin primarily modulates the inflammatory-oxidative microenvironment while supporting osteogenesis, angiogenesis, and matrix mineralization. Foundational systems combining melatonin with osteoinductive factors demonstrate synergistic pro-osteogenic and anti-resorptive effects [157], while nanocomposite scaffolds further coordinate angiogenic-osteogenic coupling in metabolically compromised defects, underscoring the value of multifunctional matrix design under diabetic or ischemic conditions [158]. A notable advance lies in pathology-responsive platforms that translate local disease cues into controlled melatonin release. Osteoporotic defects present an acidic microenvironment that triggers pH-responsive zeolitic imidazolate framework-8-based scaffolds. The consequent release of melatonin and Zn^2+^ serves to interrupt oxidative-stress-driven dysfunction while simultaneously restoring osteogenic activity [159]. Similarly, pH-labile hydrogels in infected defects enable local melatonin delivery with combined ROS-scavenging, antibacterial, and osteogenic effects (Fig. 8B) [160]. This paradigm has been further extended to inflammatory bone repair through two distinct hydrogel-based strategies. The first employs injectable hydrogels delivering melatonin-conditioned macrophage exosomes [161]. The second utilizes metalloproteinase 2-responsive adhesive hydrogels embedding melatonin-loaded mesenchymal stem cell-mimicking nanovesicles for targeted maxillofacial regeneration [162].

#### 4.4.3 Cartilage

In contrast to bone, cartilage is an avascular tissue with limited intrinsic regenerative capacity. Accordingly, melatonin-based cartilage platforms are designed primarily to preserve chondrocyte homeostasis, attenuate oxidative and inflammatory injury, and enhance ECM synthesis. A biomimetic melatonin-loaded silk fibroin/Gelatin Methacryloyl (GelMA) scaffold has been shown to promote cartilage repair by restoring mitochondrial function and improving matrix deposition [163]. Similarly, a 3D bioprinted multilayer scaffold featuring a melatonin-enriched bioactive cartilage layer further promoted cell migration, proliferation, chondrogenic differentiation, matrix deposition, and macrophage polarization [164]. In addition, pH-responsive melatonin-loaded mesoporous dopamine nanoparticles exploit the acidic osteoarthritic microenvironment while delivering both ROS-scavenging and anti-inflammatory functions [165] (Fig. 8C).

#### 4.4.4 Muscle and tendon

In soft-tissue repair, melatonin is leveraged to remodel the injury microenvironment while preserving tissue-compliant scaffold mechanics [166,174]. A representative tendon repair strategy employs a bilayer fibrous membrane that spatially decouples structural support from drug delivery (Fig. 8D1). This configuration concurrently provides mechanical reinforcement and sustained melatonin release, attenuating inflammatory and oxidative stress, promoting pro-regenerative vascular responses, and maintaining tenogenic phenotype to reduce adhesion and facilitate organized healing [166]. For skeletal muscle regeneration, a melatonin-liposome hydrogel system enables localized immuno-redox modulation within a cell-permissive matrix, significantly enhancing myofiber regeneration [167] (Fig. 8D2). Advancing toward stimulus-programmed delivery, an ROS-labile hydrogel designed for myocardial ischemia-reperfusion injury provides on-demand melatonin release under oxidative stress, suppressing inflammation, reducing cardiomyocyte apoptosis, and inducing protective autophagy to support cardiac repair [168].

#### 4.4.5 Translating non-neural tissue engineering principles to advanced MLS in PNR

The strategic development of MLS for PNR can be substantively accelerated by design principles already matured in other tissue engineering domains. This translational potential is fundamentally material-mediated. Melatonin has been successfully integrated into biomaterial categories equally central to neural repair, such as chitosan [157], GelMA/Hydroxypropyl Methylcellulose Acrylate [164], silk fibroin [163], Poly(lactide-co-glycolide) [158], and polyvinyl alcohol [168], thereby establishing a common substrate for cross-disciplinary design transfer [175,176]. Material solutions validated in bone, muscle, or dermal applications, therefore furnish directly adaptable frameworks for tailoring MLS to the specific pathophysiological demands of PNR.

A second critical design vector involves temporal release programming. Hydrophilic matrices enable diffusion-dominated rapid elution suited to early suppression of inflammation and oxidative stress, whereas hydrophobic bioresorbable polymers afford degradation-controlled sustained release aligned with the protracted course of axonal regeneration and remyelination [177]. Composite architectures integrating both mechanisms are therefore better positioned to address the full reparative continuum [178].

From this foundation, a further transferable principle emerges: converting fixed release into pathology-responsive delivery by coupling payload mobilization to lesion-associated microenvironmental cues. ROS-responsive systems exemplify this approach in the context of oxidative stress. They are typically constructed from ROS-cleavable dynamic networks, in which responsive linkages such as phenylboronic ester or boronate ester bonds undergo oxidative cleavage under high-ROS conditions. This process induces hydrogel softening, network degradation, or accelerated payload liberation [168,179,180].

Inflammation-responsive systems more often employ protease-sensitive matrices, particularly those containing matrix metalloproteinase-cleavable peptide sequences. These enable immune cell-derived enzymes to locally degrade the material network and initiate inflammation-dependent drug release [162,181]. Multiresponsive platforms integrate multiple triggers, including ROS, Ca^2+^, pH, and inflammatory signals, achieving higher temporal fidelity and lesion selectivity across the evolving stages of tissue repair [159,182,183].

Melatonin stands to benefit substantially from this design logic. By coupling release behavior to the evolving immuno-redox landscape of the injured nerve, such systems may better preserve the temporal coherence of repair while reducing the risks of mistimed exposure. In this way, pathology-responsive MLS could offer a more biologically attuned framework for converting melatonin's pleiotropic activity into durable regenerative benefit in PNR. This adaptive paradigm may be particularly valuable in reconciling the shifting concentration requirements associated with acute cytoprotection, immunomodulation, and later-stage regenerative support.

Design concepts from non-neural melatonin delivery platforms remain relevant to PNR, encompassing stimulus-sensitive release mechanisms, reservoir compartmentalization, and sequential delivery programming. However, their direct transfer must be mediated by nerve-specific engineering imperatives: mechanical compliance, sustained guidance stability, controllable degradation, and neurovascular compatibility. Adaptive, nerve-centric architectural solutions therefore supersede the unmodified adoption of extra-neural platform blueprints.

### 4.5 MLS developmental trajectory and emerging trends

**Fig. 9:**
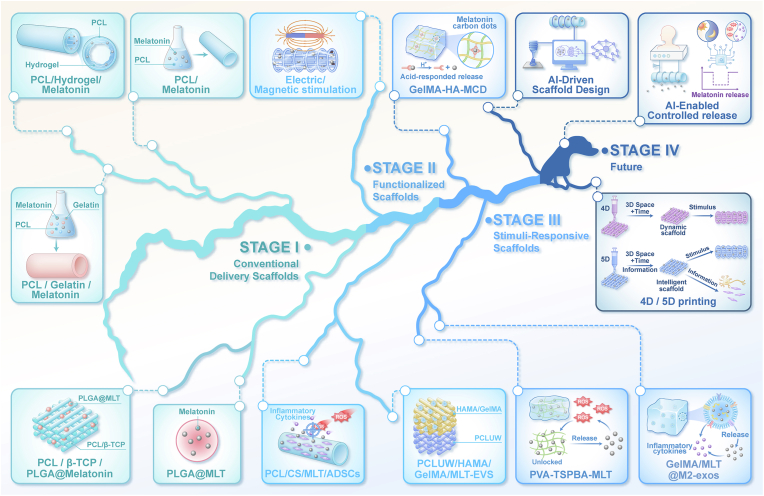
Amazon River–inspired river-flow schematic summarizing the four-stage evolution of melatonin-enabled tissue-engineered scaffolds. Stage I: conventional delivery scaffolds that prolong local melatonin availability. PLGA@MLT: Poly(lactide-co-glycolide)@melatonin. PCL/β-TCP/PLGA@Melatonin: PCL/β-tricalcium phosphate/PLGA@melatonin [158]. Stage II: functionalized scaffolds that integrate melatonin delivery with added instructive functions. PCLUW/HAMA/GelMA/MLT-EVs: PCL/Hydroxyapatite ultralong nanowire/hyaluronic acid/gelatin methacryloyl/Melatonin-extracellular vesicles [164]. Stage III: stimuli-responsive scaffolds that couple releases to endogenous pathological cues. GelMA-HA-MCD: GelMA-oxidized hyaluronic acid hydrogel-melatonin carbon dot [160]. PVA-TSPBA-MLT: polyvinyl alcohol- N1-(4-boronobenzyl)-N3-(4-boronophenyl)-N,1 N,1 N3,N3-tetramethylpropane-1,3-diaminium - mealtonin [168]. GelMA/MLT@M2 exos: GelMA/melatonin-loaded M2 macrophage exosomes [161]. Stage IV: future intelligent platforms featuring programmable, feedback-regulated release supported by AI-enabled design and advanced manufacturing concepts.

The evolution of MLS in tissue engineering has progressed from simple encapsulation strategies toward sophisticated, integrated platforms. In these advanced systems, release kinetics are deliberately engineered as an intrinsic component of overall scaffold functionality. This developmental trajectory can be categorized into three distinct design stages, corresponding to a shift in priorities: initial focus on local drug retention, subsequent integration of multi-modular functional coupling, and ultimately, the achievement of endogenous cue-responsive regulation (Fig. 9). Notably, although numerous exemplary systems originate from applications outside the peripheral nerve field, their foundational design principles retain significant portability. These principles can thus be strategically adapted to inform and accelerate MLS development specifically for PNR.

#### 4.5.1 Stage I: conventional delivery scaffolds

The inaugural design generation of MLS was defined by a singular engineering imperative: maximizing local drug bioavailability and extending lesional melatonin retention. Physical encapsulation within biodegradable polymeric carriers, including conduits, hydrogel composites, and microsphere systems, constituted the predominant implementation strategy, expressly conceived to circumvent the rapid clearance and poor lesion partitioning that limit conventional melatonin delivery [14,144,158]. Release kinetics remained governed by passive diffusion and carrier degradation, channeling optimization toward prolonged elution and burst suppression. Functionally, these platforms served as stationary pharmacological depots, achieving sustained local melatonin elevation sufficient to confer measurable anti-inflammatory and neuroprotective efficacy. Yet the design architecture remained intrinsically one-dimensional, affording no capacity for dynamic therapeutic programming or stage-adaptive release regulation.

#### 4.5.2 Stage II: functionalized scaffolds

Increasing appreciation of regenerative microenvironment dynamics exposed the therapeutic insufficiency of sustained melatonin release alone, driving the evolution of scaffolds that couple drug delivery with complementary instructive modalities. One predominant strategy integrates electroconductive or magnetoresponsive components, enabling concurrent melatonin-mediated immuno-redox modulation and exogenous physical stimulation [143,145]. Parallel designs incorporate biological effectors, including stem cells or exosome carriers, to furnish integrated cellular and paracrine support [148,164]. Within these advanced constructs, melatonin is repositioned from an isolated payload to a coordinated element within a multifactor system wherein material properties, physical signaling, and biological cues are jointly engineered to coherently regulate inflammation, cell survival, and tissue organization. Yet regulatory capacity remains constrained by reliance on predefined functional modules and dependence on external actuation.

#### 4.5.3 Stage III: stimuli-responsive scaffolds

As an advanced delivery paradigm, stimuli-responsive melatonin scaffolds dynamically couple release behavior to the evolving pathology of the injury microenvironment. These systems eschew fixed, preprogrammed kinetics by sensing endogenous cues, including inflammatory activation, oxidative stress, and pH dysregulation, thereby affording spatiotemporally synchronized drug exposure [160,168]. Applications in peripheral nerve engineering, while still emergent, instantiate a decisive conceptual shift toward feedback-guided therapeutic regulation. This paradigm constitutes the essential engineering bridge linking current scaffold architectures to next-generation intelligent platforms endowed with autonomous delivery precision and therapeutic selectivity.

#### 4.5.4 Stage IV: intelligent, programmable, and feedback-regulated scaffolds

Next-generation MLS are shifting from optimizing isolated release kinetics toward implementing an integrated control logic. This framework co-designs sensing, computation, and actuation to enable context-aware, feedback-governed delivery. Artificial intelligence serves as a foundational design engine, accelerating material selection, structural refinement, and release profile prediction to enhance both development efficiency and system reproducibility [184]. Clinically, predictive modeling facilitates prognosis forecasting and patient stratification, guiding personalized scaffold selection, dosing, and monitoring [185]. Integration of microenvironmental sensors with delivery modules permits direct detection of local pathological cues, enabling dynamic tuning of melatonin release to align with the phased repair process [186].

3D printing has emerged as a powerful biofabrication strategy in peripheral nerve tissue engineering. This approach enables precise construction of customized nerve-guidance scaffolds featuring complex geometries, branched architectures, multichannel organization, and spatially integrated functional cues [187,188]. Although 3D printing-based strategies have thus far been only sparsely explored in melatonin-based scaffold systems for PNR, ongoing advances in multimaterial printing, hierarchical microstructural control, and spatially defined bioactive loading may open new possibilities for the development of more biomimetic and therapeutically coordinated platforms.

Extending beyond the structural precision afforded by 3D printing, concurrent advances in four-dimensional (4D) printing have introduced scaffold systems capable of time-dependent geometric and functional evolution [189,190]. In contrast to static scaffolds, 4D-printed systems are built from stimuli-responsive materials that undergo programmed transformations in response to thermal, chemical, optical, magnetic, or biochemical cues, thereby introducing a temporal dimension to scaffold behavior and drug delivery [191]. In melatonin-based scaffold engineering, this dynamicity may enable stage-specific and potentially circadian-aware melatonin release through adaptive changes in scaffold conformation, porosity, mechanical properties, and diffusion pathways. Such adaptive changes allow therapeutic exposure to better align with the evolving microenvironment of PNR and, in principle, with favorable circadian windows [192].

Five-dimensional (5D) printing remains a non-standardized extension of 4D printing that incorporates an additional information dimension and may further enhance structural complexity, geometric conformity, and functional integration [193]. Although still largely conceptual in peripheral nerve tissue engineering, this approach may provide a useful framework for more adaptive and hierarchically integrated scaffold design.

## 5 Engineered melatonin delivery for non-traumatic PN

Melatonin addresses the convergent pathophysiology of DPN and CIPN through coordinated modulation of immune activation, oxidative stress, and circadian rhythm. Despite this mechanistic relevance, tissue-engineered delivery systems for non-traumatic neuropathies remain markedly underdeveloped, with current approaches largely adapted from PNI-dedicated paradigms. This section therefore first delineates melatonin's key biological roles in DPN and CIPN. It subsequently reviews existing delivery formats suitable for these contexts, emphasizing regimens compatible with repeated administration under the constraints of diffuse lesions and chronic progression. Building on this foundation, illustrative melatonin-loading strategies from adjacent tissue engineering fields are examined to extract transferable design principles and to propose a materially and architecturally grounded framework for developing melatonin-enabled systems expressly targeting DPN and CIPN.

### 5.1 Mechanistic interventional targets of melatonin for non-traumatic PN

In contrast to surgically reparable traumatic nerve gaps, DPN and CIPN are characterized by diffuse, chronic dysfunction distributed throughout the neural microenvironment. Sustained immuno-redox stress, amplified by circadian dysregulation, progressively disrupts sensory processing and accentuates diurnal fluctuations in stress tolerance and pain perception. Within this context, melatonin emerges as a microenvironment-active, time-coupled therapeutic agent capable of synchronizing immuno-redox control with circadian biology. This section delineates the mechanistic rationale for melatonin-based intervention in DPN and CIPN. It further establishes precise exposure control and temporal alignment as fundamental design imperatives for any delivery strategy seeking therapeutic efficacy beyond symptomatic management.

#### 5.1.1 Endogenous melatonin axis and circadian vulnerability in non-traumatic PN

Evidence directly links dysregulated endogenous melatonin secretion to the pathogenesis of DPN. Clinically, DPN patients exhibit significantly reduced urinary 6-sulfatoxymelatonin levels compared with healthy individuals [194]. Genetic analyses further substantiate this association; the rs2119882 G allele confers protection, whereas the rs13140012 polymorphism increases DPN risk approximately fivefold under a recessive model [195]. Circadian disruption analogously contributes to CIPN. Paclitaxel and related agents suppress the rhythmic expression of core clock genes, including Arntl, Nr1d1, and Per1, within the suprachiasmatic nucleus [196]. This central circadian impairment likely perturbs physiological melatonin secretion and may indirectly amplify neuroinflammatory and oxidative stress responses during chemotherapy.

#### 5.1.2 Multitarget mechanisms of melatonin in non-traumatic PN

Melatonin exerts therapeutic effects in DPN and CIPN primarily through coordinated anti-inflammatory and antioxidant actions that directly counter the immuno-redox dysregulation driving sensory dysfunction [197,198]. Mechanistic attention has increasingly converged on the dorsal root ganglion (DRG), where mitochondrial stress in sensory neurons serves as a pivotal pathogenic event [199]. A recently defined glia-neuron support mechanism involves functional mitochondrial transfer from satellite glial cells to neurons via tunneling nanotubes, a process critical for neuronal metabolic homeostasis [199]. Melatonin significantly potentiates this tunneling nanotube-mediated intercellular transfer, thereby enhancing mitochondrial delivery to stressed neurons and reinforcing organelle resilience [200]. These findings collectively position melatonin's benefits beyond generalized immuno-redox modulation; it specifically attenuates neurodegeneration and pain in DPN and CIPN by reinforcing DRG mitochondrial homeostasis through facilitated intercellular organelle support.

### 5.2 Engineered delivery systems for non-traumatic PN

The design of delivery platforms for DPN and CIPN is fundamentally constrained by a distinct pathological topography: diffuse lesions, persistent microenvironmental stress, and a clinical preference for repeatable, minimally invasive intervention over structural reconstruction. Given limited tissue accessibility and rapid systemic clearance, prevailing strategies accordingly prioritize non-implantable or minimally invasive formats engineered to sustain effective local drug exposure.

#### 5.2.1 Engineered delivery systems for DPN

**Fig. 10:**
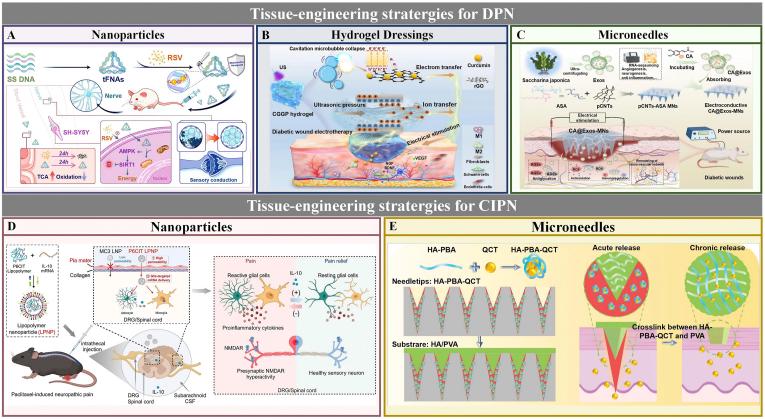
Tissue-engineering strategies for DPN and CIPN. A-C: Representative tissue-engineering design in DPN. A. Nanoparticle-based delivery. Reproduced with permission from Ref. [201]. B. Hydrogel dressing systems. Reproduced with permission from Ref. [202]. C. Microneedles patches. Reproduced with permission from Ref. [203]. D-E: Representative tissue-engineering design in CIPN. D. Nanoparticle-based delivery. Reproduced with permission from Ref. [204]. E. Microneedles patches. Reproduced with permission from Ref. [205].

The management of DPN demands material platforms that adapt to its evolving pathology, from diffuse subcutaneous lesions to open ulcers. In early, non-ulcerated stages, the absence of structural discontinuity favors minimally invasive, repeatable injectable systems. Nanoscale carriers, including self-assembling nucleic acid nanostructures (Fig. 10A) and stimuli-responsive inorganic platforms, enhance local bioavailability and couple antioxidant activity with pro-regenerative signaling [201,206]. These strategies collectively prioritize distal tissue targeting, repeatable administration, and sustained local retention.

With progression to ulceration, the therapeutic challenge expands. Foot and hand ulcers create a complex niche wherein inflammation, oxidative stress, hypoxia, and infection converge to exacerbate neural injury and pain [202]. Integrated wound management therefore necessitates dressings capable of simultaneous immuno-redox modulation and infection control. Hydrogel composites, with their tunable hydration and release kinetics, are ideally suited (Fig. 10B); representative designs include environmentally responsive antioxidant delivery systems, conductive networks for multimodal stimulation, and ROS-labile nanocomposites combining on-demand antioxidant release with sonodynamic antibacterial therapy [4,202,207].

Extending beyond passive dressings, microneedle platforms enable active, minimally invasive transdermal delivery to superficial and peri-wound tissues [208]. Stimulus-responsive variants afford on-demand control through integrated sensing and triggered release modules [208]. Conductive patches constitute a parallel advancement, employing electrically active polymer composites for exosome-mediated, electro-assisted therapeutic delivery (Fig. 10C) [203].

#### 5.2.2 Engineered delivery systems for CIPN

CIPN shares key clinical features with early-stage DPN: diffuse, length-dependent sensory dysfunction without ulceration. Pathology may ascend from distal terminals to proximal sensory compartments, mandating minimally invasive, repeatable delivery strategies with sufficient tissue penetrance and precise kinetic control.

Nanoparticle carriers are particularly suited to these demands. mRNA-loaded nanoparticles achieve transdural transport and preferential DRG accumulation for compartment-targeted immuno-glial modulation (Fig. 10D) [204]. Macrophage-targeted antioxidant nanoparticles, engineered for inflammatory homing, concurrently act as ROS-scavenging nanozymes to mitigate oxidative injury [209].

Microneedle systems provide a programmable transdermal alternative. A bilayer patch designed for biphasic release delivers rapid initial availability followed by sustained elution, supporting both immediate intervention and prolonged local exposure (Fig. 10E) [205].

### 5.3 Engineering melatonin delivery systems from non-traumatic PN translational blueprints

Melatonin-functionalized tissue engineering platforms purpose-built for non-traumatic PN remain conspicuously underdeveloped. Yet nanoparticle, hydrogel, and microneedle systems originally engineered for other pathological contexts already encapsulate transferable delivery paradigms directly relevant to DPN and CIPN management. These extant platforms therefore constitute a strategic translational reservoir through which proven delivery architectures can be systematically redeployed to redress the unmet therapeutic requirements of DPN and CIPN. This established knowledge base thereby affords a rationally anchored foundation for subsequent platform refinement and disease-specific customization. Representative transferable platforms and design strategies are summarized in Table 6.

**Table 6:** Transferable MLS from other regenerative contexts for non-traumatic PN.

Platform	Type	Tissue/Organ	Melatonin delivery mode	Ref
M-Ang2-EVs	Nanoparticle	spinal cord	Peptide-mediated targeted delivery	[210]
CXCR2-MM@PLGA/MT		liver	Receptor-mediated targeted delivery	[211]
C/AXG/AGNP/MELT	Hydrogel dressings	skin	Diffusion-controlled release	[212]
PEG@MT				[213]
MT-PAM				[214]
MT-SF MN	Microneedles			[215]
SOP			Wireless electro-triggered release	[216]

**Abbreviation:**M-Ang2-EVs: Melatonin-preconditioned M2 microglia-derived Angiopep-2-modified extracellular vesicles; CXCR2-MM@PLGA/MT: Genetically engineered macrophage membrane-coated PLGA nanoparticles loaded with melatonin; C/AXG/AGNP/MELT: Collagen/aminated xanthan gum/bio-capped Ag-nanoparticles/MELT hydrogel; PEG@MT: Melatonin-loaded injectable polyethylene glycol-based hydrogel; MT-PAM: Melatonin-polyacrylamide adhesive hydrogel; MT-SF MN: L-proline-assisted silk fibroin microneedle patch loaded with melatonin; SOP: Spatiotemporal on-demand patch loaded with melatonin.

#### 5.3.1 Nanoparticles

Nanoparticle platforms represent a broadly transferable strategy for spatiotemporally controlled, lesion-biased drug delivery under biological barriers and inflammatory constraints. Their translational relevance is particularly pronounced in central nervous system injury. In this context, a major impediment to therapeutic implementation lies in enabling bioactive cargos to penetrate the blood-brain barrier or blood-spinal cord barrier. Against this backdrop, two strategies have emerged as especially compelling. The first involves immune cell-mediated delivery [217]. The second employs receptor- or ligand-directed targeting modifications [218]. The growing recognition that blood-spinal cord barrier re-establishment constitutes an integral component of tissue repair has further strengthened the rationale for vascular-targeted receptor-ligand approaches. These approaches are designed to engage nascent barrier-associated structures [219,220].

**Fig. 11:**
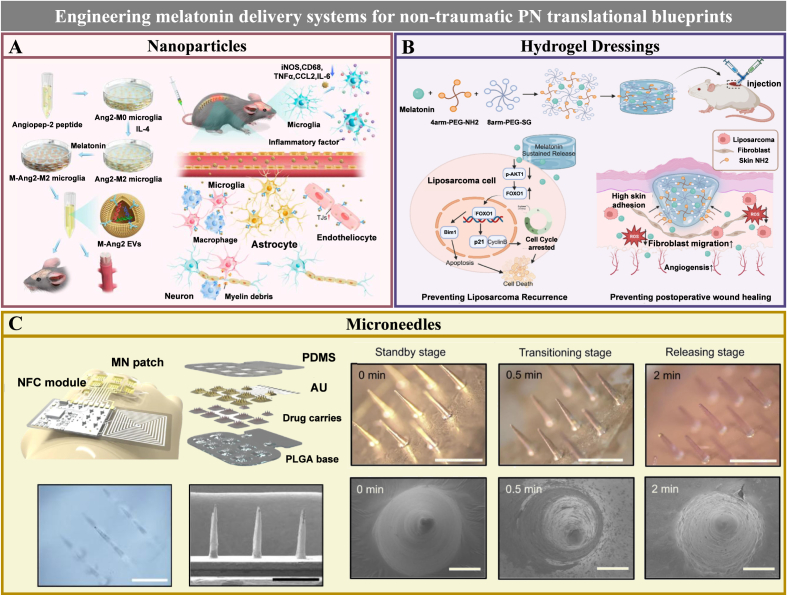
Transferable tissue-engineering designs for melatonin in DPN and CIPN. A. Design example of melatonin incorporated into nanoparticle-based carriers. Reproduced with permission from Ref. [210]. B. Design example of melatonin integrated with a hydrogel dressing for local, sustained delivery. Reproduced with permission from Ref. [213]. C. Design example of melatonin combined with microneedle platforms to enable minimally invasive, spatiotemporally controlled administration. Reproduced with permission from Ref. [216].

Melatonin-related applications have already begun to embody this design logic. Peptide-modified M2 macrophage-derived extracellular vesicles have been employed to deliver melatonin to spinal cord injury sites (Fig. 11A) [210]. Comparable targeting principles have also been demonstrated in liver injury, where neutrophil chemokine-modified macrophage membranes endowed melatonin-loaded microspheres with affinity for hepatocytes and macrophages [211]. These advances suggest that cell-mimetic and lesion-adaptive targeting strategies may furnish a transferable framework for precision melatonin delivery in non-traumatic PN, enabling more selective engagement of pathogenic or reparative cellular populations within the diseased nerve microenvironment.

#### 5.3.2 Hydrogel dressings

Although melatonin-loaded hydrogel dressings remain underexplored in DPN management, extensive cutaneous wound research validates their potential to beneficially reshape the wound microenvironment via coordinated anti-inflammatory and antioxidant activities. Hybrid biopolymer-nanoparticle composites enable co-delivery of melatonin with complementary bioactive agents, functioning as immunomodulatory dressings that actively condition the wound bed [212]. Adhesive hydrogel architectures address the need for conformal contact and prolonged retention on mobile skin; melatonin-integrated multi-arm polyethylene glycol networks enhance wound closure and angiogenic remodeling (Fig. 11B) [213]. For highly mobile anatomical sites, mechanically compliant, bandage-reinforced hydrogels ensure stable, sustained melatonin delivery under repetitive flexing [214].

#### 5.3.3 Microneedles

Microneedle-mediated melatonin delivery has advanced from sustained local retention toward spatiotemporally precise, triggerable administration, meeting the need for repeatable, minimally invasive intervention. Early designs achieved prolonged transdermal release; protein-based microneedle patches delivered sustained melatonin exposure over hours with high cumulative elution and demonstrated efficacy in a preclinical sleep model [215]. Recent innovation has shifted toward active, on-demand control. A representative platform employs polymer microneedles with an electrically responsive metallic coating; low-voltage stimulation triggers controlled release through electrochemical coating disruption. Intracranial deployment confirmed precise, programmable melatonin delivery to targeted brain regions, establishing triggerable microneedle architectures as a precision platform for neurological applications (Fig. 11C) [216]. Such a triggerable system may also be well suited for circadian-timed delivery, as the ability to apply stimulation at predefined time points enables synchronization of melatonin release with circadian rhythms.

#### 5.3.4 Transferable design logic for MLS in non-traumatic PN

Taken together, the transferable design logic for melatonin-based systems in non-traumatic PN can be distilled into a pathology-topography-guided, minimally invasive, and programmable engineering paradigm. Platform selection should be governed not merely by the objective of prolonging local retention, but by lesion distribution, tissue accessibility, and disease stage. Nanoparticle systems are particularly well suited to deep, diffuse, and cell-directed intervention. They may be further refined through ligand-functionalization to enhance lesion-selective accumulation and cellular uptake. Mannan-based modification enables macrophage targeting, while neurotropic peptides facilitate nerve-associated delivery [221,222]. By contrast, hydrogel dressings are more appropriately configured for ulcerative lesions with overt tissue defects. In such applications, conformal coverage, sustained local residence, and microenvironment-responsive melatonin release can be integrated to restore hydration and mitigate oxidative and inflammatory stress [213]. Microneedle platforms offer a complementary minimally invasive route for localized and spatially defined administration. They also provide the additional advantage of externally regulated and temporally programmable release [216]. Ultimately, the central transferable principle lies not in the adoption of any single carrier format, but in the rational integration of lesion-matched platform selection, responsive release behavior, targeting design, and clinically feasible dosing control into a unified melatonin delivery framework for non-traumatic PN.

## 6 From bench to bedside translation of melatonin scaffolds

Despite substantial progress in peripheral nerve tissue engineering and melatonin-based PNR, their convergence into clinically viable therapies remains underdeveloped. Current interventions target structural reconstruction or symptomatic relief, whereas melatonin platforms have not advanced beyond preclinical investigation. This section analyzes the translational landscape by evaluating existing engineered platforms, the clinical status of melatonin, and key convergence barriers. From this analysis, we identify critical bottlenecks and propose practical priorities to guide the development and clinical translation of integrated melatonin-tissue engineering strategies.

### 6.1 Clinically available tissue-engineered products for PN

The clinical management of PN increasingly incorporates tissue-engineered products selected according to disease etiology and therapeutic goals [223]. This section catalogs clinically available biomaterial platforms for PNI and examines analogous strategies applied to, or translatable for, non-traumatic PN.

#### 6.1.1 Engineered therapeutic strategies for PNI

Clinically translated biomaterials for PNR comprise biologically derived and synthetic polymer systems. Collagen-based scaffolds, the most extensively utilized natural platform, offer high biocompatibility and low immunogenicity [224]. Approved collagen conduits now incorporate internal architectures to enhance guidance and mechanical performance [225]; collagen membranes and decellularized ECM products are widely applied as protective wraps [223,226]. Chitosan provides a second biogenic platform valued for predictable degradation and antibacterial activity; approved conduits achieve autograft-comparable outcomes for short gaps, with membrane formats facilitating nerve protection [49]. Hydrogel systems, including approved alginate–hyaluronic acid composites, provide tissue compliance as suture-free protectors; injectable variants improve anatomical conformability [223,227].

Synthetic conduits afford manufacturing consistency, mechanical tunability, and controlled degradation. Polyglycolic acid conduits, established early devices, demonstrate reliable safety and efficacy for short defects [228]. Poly(L-lactide-co-caprolactone) offers adjustable degradation but faces adoption constraints related to handling and cost [229]; newer copolymers target improved hydrophilicity and degradation profiles, though clinical validation is pending [223].

#### 6.1.2 Engineered therapeutic strategies for non-traumatic PN

Biomaterials and tissue-engineered products specifically targeting non-traumatic PN are entirely absent from clinical practice. For DPN, no approved intervention is engineered to preserve nerve function through active microenvironmental modulation. Available products instead address diabetic foot ulcers, prioritizing wound coverage and soft tissue replacement over neural repair [223]. These dressings maintain a moist environment and promote closure, yet their functional scope remains structural, with limited capacity to redress chronic inflammation, oxidative stress, or microvascular dysfunction. Neuroprotection and disease modification are thus not integral to their design.

The translational landscape for CIPN is even less advanced. No tissue-engineered construct or biomaterial-enabled delivery platform is approved for CIPN management. This gap is further underscored by the regulatory status of capsaicin patches, approved for other neuropathic pain conditions but not explicitly indicated for CIPN [230].

### 6.2 Clinical landscape of melatonin-based therapeutic applications

The clinical translation of melatonin for PN is constrained by three interrelated barriers: suboptimal pharmacokinetics, heterogeneous regulatory status, and insufficient clinical validation. Melatonin exhibits poor bioavailability and rapid hepatic clearance [231]; its regulatory classification differs between the United States and European Union [232]. Clinical evidence remains largely preclinical, with human studies absent for traumatic injury and limited to adjunctive use or small trials in non-traumatic neuropathies [233,234]. A fundamental bottleneck is the failure of systemic administration to achieve therapeutic concentrations within peripheral nerve tissue [233]. Integration of melatonin into clinically approved PNR devices has not been pursued. Consequently, while tissue-engineered local delivery represents a compelling strategic alternative, its advancement requires rigorous clinical validation of safety, efficacy, and dosing.

### 6.3 Translational challenges and bottlenecks of MLS for PN

The clinical translation of MLS for PNR is constrained by interdependent manufacturing, design, evidence, and regulatory challenges. Formulation variability across melatonin dose, carrier materials, and scaffold properties impedes reproducible benchmarking; this is compounded by drug stability concerns and Good Manufacturing Practice compliance demands [235]. A critical design limitation is the static release profile of current systems, which fails to align with the stage-specific biology of regeneration. Stimulus-responsive concepts remain unproven in long-term safety and translational robustness. The clinical evidence base is severely underdeveloped, lacking large-scale trials against surgical standards or patient-centered outcomes. These challenges converge within a stringent regulatory framework. Classification as drug-device combination products necessitates integrated evaluation of scaffold performance and drug delivery, imposing greater regulatory complexity than for standalone devices or pharmaceuticals [236].

## 7 Conclusion and prospect

### 7.1 Conclusion

PN comprises a spectrum of disorders converged by a unifying pathological substrate: microenvironmental destabilization perpetuated by chronic inflammation, redox imbalance, and circadian disruption. Contemporary symptomatic interventions inadequately engage these core mechanistic drivers. Melatonin presents a distinctive multi-target pharmacological strategy, integrating immuno-modulatory, redox-buffering, and chronobiotic actions within the neural niche. This review critically appraises the therapeutic rationale for melatonin in PN. Framed within the MLS paradigm, it traces the generational succession of scaffold architectures for PNR, extracts transferable design logic from adjacent tissue engineering fields to inform non-traumatic PN strategies, and consolidates the principal translational bottlenecks. These barriers span standardization deficits, scalable manufacturing constraints, clinical workflow integration challenges, and the regulatory complexity inherent to drug-device combination products. The strategic advancement of scalable, clinically anchored biofabrication positions melatonin-integrated systems for transition from preclinical promise toward deployable therapeutic solutions capable of redefining the interventional landscape for PN.

### 7.2 Prospects

Notwithstanding the substantial progress achieved thus far, the further maturation of MLS for PN remains contingent upon overcoming persistent challenges in chronobiological coordination, delivery precision, and translational robustness. Even so, continuing advances in biomaterials engineering are steadily expanding the conceptual and practical horizon of this field.

#### 7.2.1 Circadian-synchronized and stage-specific release

The circadian nature of melatonin secretion bears direct relevance to its therapeutic application, yet this dimension has not been explicitly incorporated into the design logic of existing melatonin-based scaffold systems. Looking ahead, externally actuated tissue-engineered delivery systems responsive to stimuli such as ultrasound, magnetic fields, electrical cues, or light may offer a feasible strategy for circadian-synchronized melatonin release. Stimulation can be applied at deliberately defined time points to achieve temporally controlled administration [[237], [238], [239]]. Beyond such externally controlled strategies, a potentially more transformative direction lies in endogenous clock-driven delivery systems. Coupling core circadian genes, such as Period2, with melatonin-related biosynthetic genes and introducing these circuits into pluripotent stem cell-derived engineered tissues or organoid-like constructs may enable harnessing intrinsic circadian gene oscillations to drive the timed synthesis and release of melatonin without continuous external intervention [240]. Beyond direct gene-level coupling, circadian rhythmicity may also be encoded at the cis-regulatory level by integrating clock-responsive promoter elements into synthetic circuits, thereby enabling tunable oscillatory transcription with defined phase characteristics [241]. At a further stage of translational integration, wearable biosensing, programmable actuation systems, and artificial intelligence-assisted chronotherapy modeling may enable individualized rhythm-synchronized delivery, with stimulation schedules dynamically optimized according to patient-specific circadian patterns and lesion evolution [242,243]. Such closed-loop platforms could ultimately shift melatonin administration from preprogrammed release toward adaptive, biologically informed intervention.

#### 7.2.2 Cell-selective targeting and hierarchically programmed dosing

Across the regenerative continuum, distinct cellular populations do not share identical melatonin requirements. Acute inflammatory control may require relatively high local exposure, whereas Schwann cells, endothelial cells, and neurons are more effectively engaged within lower or intermediate concentration windows during later repair. This divergence points toward hierarchically programmed, cell-selective dosing rather than uniform release [244]. A plausible solution combines temporally staged delivery with melatonin-loaded nanocarriers bearing surface-encoded recognition motifs, such as antibodies, peptides, aptamers, or biomimetic membrane ligands [210,245,246]. In such a framework, an early inflammatory-cell-targeting module could preferentially direct melatonin to macrophage-enriched compartments, followed by a delayed neuroglial-targeting module that sustains a more regeneration-permissive exposure around Schwann cells and neurons. Such a strategy enables compartment- and stage-specific melatonin exposure, thereby offering a more elegant means of reconciling competing concentration demands within the injured nerve niche.

#### 7.2.3 Clinically translatable platform design

Future melatonin-based scaffold development should be oriented not solely toward escalating functional sophistication, but equally toward the practical imperatives of manufacturing reproducibility, sterilization robustness, surgical operability, and regulatory tractability. Within such a translational framework, bionic scaffolds may constitute a particularly compelling route toward clinical implementation [247]. Relative to highly intricate multicomponent intelligent systems, these platforms are generally more amenable to standardized fabrication, rely on less compositionally complex material architectures, and permit the integration of localized drug delivery with structural guidance without materially increasing operative burden. Representative configurations include aligned nanofibrous topographies, multichannel biomimetic constructs, and nerve membrane-mimetic architectures, all of which recapitulate salient structural attributes of native neural tissue and thereby facilitate directed axonal regeneration [188,248,249]. Structural biomimicry alone, however, is rarely sufficient to recalibrate the post-injury microenvironment, particularly the inflammatory and oxidative disequilibrium that constrains effective repair. In this respect, melatonin incorporation may offer a strategically streamlined yet biologically enriched solution, enabling the convergence of structural guidance and local microenvironmental modulation while preserving translational practicality.

## CRediT authorship contribution statement

**Mouyuan Sun:** Conceptualization, Funding acquisition, Methodology, Project administration, Software, Validation, Writing – review & editing. **Xuankai Fan:** Data curation, Investigation, Resources, Writing – original draft. **Yaxian Luo:** Formal analysis, Validation, Visualization, Writing – original draft. **Luying Qin:** Validation, Visualization. **Shuangyang Li:** Formal analysis, Investigation. **Jingyu Zhang:** Writing – review & editing. **Zhixu He:** Software. **Lianjie Peng:** Validation. **Tao Qiu:** Conceptualization. **Tian Zhang:** Methodology. **Huiming Wang:** Funding acquisition, Supervision. **Mengfei Yu:** Formal analysis, Funding acquisition, Project administration, Supervision, Writing – review & editing.

## Declaration of Competing Interest

All authors declare no known competing financial interests or personal relationships that could have appeared to influence the work reported in this article.

## Data Availability

Data will be made available on request.
