# Supplementary material for: Harnessing spatiotemporal melatonin delivery from engineered platforms for targeted microenvironment remodeling in peripheral neuropathy
Source: Mater Today Bio. 2026 Apr 22;38:103144. doi: 10.1016/j.mtbio.2026.103144 (PMC13145392; doi:10.1016/j.mtbio.2026.103144)
Supplement: Multimedia component 1 [file mmc1.docx]

**Supporting Information of**

**Harnessing Spatiotemporal Melatonin Delivery from Engineered Platforms for Targeted Microenvironment Remodeling in Peripheral Neuropathy**

Mouyuan Sun^1^#*, Xuankai Fan^1^#, Yaxian Luo^1^#, Luying Qin^1^, Shuangyang Li^1^, Jingyu Zhang^1^, Zhixu He^1^, Lianjie Peng^1^, Tao Qiu^1^, Tian Zhang^1^, Huiming Wang^1^, Mengfei Yu^1^*

1. Stomatology Hospital, School of Stomatology, Zhejiang University School of Medicine, Zhejiang Provincial Clinical Research Center for Oral Diseases, Zhejiang Key Laboratory of Oral Biomedical, China, Hangzhou, 310000

# These authors contributed equally

* Corresponding authors: Mengfei Yu (yumengfei@zju.edu.cn), Mouyuan Sun (sunmouyuan777@zju.edu.cn)

**1. Meta analysis**This systematic review and network meta-analysis was registered with PROSPERO (CRD420261346501) and conducted in accordance with the PRISMA 2020 statement. A comprehensive literature search was independently performed by two investigators across four electronic databases, including PubMed (MEDLINE), Embase, Web of Science, and Cochrane CENTRAL, for English-language studies published from January 1, 2000, to December 1, 2025. Because the review addressed three distinct clinical settings, separate search strategies were developed for peripheral nerve injury (PNI), diabetic peripheral neuropathy (DPN), and chemotherapy-induced peripheral neuropathy (CIPN).
For the PNI analysis, the search strategy was: ("peripheral nerve injury" OR "peripheral nerve injuries" OR "peripheral nerve repair" OR "peripheral nerve defect" OR "digital nerve injury" OR "digital nerve injuries" OR "digital nerve repair" OR "digital nerve defect" OR "digital nerve defects" OR "digital nerve transection") AND ("autograft" OR "autografts" OR "autologous nerve graft" OR "nerve autograft" OR "suture" OR "neurorrhaphy" OR "end-to-end neurorrhaphy" OR "end to end neurorrhaphy" OR "synthetic conduit" OR "synthetic conduits" OR "nerve conduit" OR "nerve conduits" OR "nerve guidance conduit" OR "nerve guidance conduits" OR "nerve tube" OR "nerve tubes" OR "decellularized allograft" OR "decellularized allografts" OR "decellularized nerve allograft" OR "decellularized nerve allografts"). The primary outcome for this analysis was sensory recovery assessed by static two-point discrimination (S2PD). Animal studies, reviews, conference abstracts, dissertations or theses, duplicate publications, and studies with missing outcome data were excluded. A total of 29 studies met the eligibility criteria, of which 7 two-arm or multi-arm comparative studies were ultimately included in the network meta-analysis.

For the DPN analysis, the search strategy was: ("diabetic peripheral neuropathy" OR "diabetic neuropathy" OR "diabetic polyneuropathy" OR "painful diabetic neuropathy" OR "painful diabetic peripheral neuropathy" OR "diabetic neuropathic pain" OR "diabetic peripheral neuropathic pain" OR "PDN" OR "DPN") AND ("pregabalin" OR "Lyrica" OR "duloxetine" OR "Cymbalta" OR "gabapentin" OR "Neurontin" OR "capsaicin" OR "capsaicin patch" OR "Qutenza" OR "high-concentration capsaicin"). The primary outcome was the proportion of patients achieving at least 50% pain reduction. Animal studies, reviews, conference abstracts, dissertations or theses, duplicate publications, and studies with missing outcome data were excluded. A total of 22 studies met the eligibility criteria, of which 19 two-arm or multi-arm comparative studies were included in the final network meta-analysis.

For the CIPN analysis, the search strategy was: ("chemotherapy-induced peripheral neuropathy" OR "chemotherapy induced peripheral neuropathy" OR "chemotherapy-induced neuropathy" OR "chemotherapy induced neuropathy" OR "painful chemotherapy-induced peripheral neuropathy" OR "painful chemotherapy induced peripheral neuropathy" OR "chemotherapy-induced peripheral neuropathic pain" OR "chemotherapy-induced neuropathic pain" OR "chemotherapy induced neuropathic pain" OR "chemotherapy-related peripheral neuropathy" OR "CIPN") AND ("duloxetine" OR "gabapentin" OR "pregabalin" OR "amitriptyline" OR "capsaicin" OR "capsaicin patch" OR "capsaicin 8%") The primary outcome was the mean reduction in pain intensity scores on a 10-point scale. Animal studies, reviews, conference abstracts, dissertations or theses, duplicate publications, and studies with missing outcome data were excluded. Owing to limited data availability, standard deviations for some studies were derived from the reported 95% confidence intervals using standard formulas; when imputation of change-score variance was required, a correlation coefficient of 0.5 was assumed. A total of 5 studies met the eligibility criteria, all of which were two-arm or multi-arm comparative studies and were ultimately included in the network meta-analysis.
A Bayesian network meta-analysis was performed using R software (version 4.3.0) with the gemtc and rjags packages, applying random-effects models to estimate relative effects and rank treatments. Methodologically, we constructed a connected evidence network and used the Markov Chain Monte Carlo (MCMC) algorithm for parameter estimation, running four chains for 50,000 iterations, with a 20,000-iteration warm-up period to ensure convergence. Convergence of the Bayesian network meta-analyses for PNI, DPN, and CIPN was assessed using Markov chain Monte Carlo (MCMC) diagnostics, including trace plots, posterior density plots, and Brooks-Gelman-Rubin shrink factor plots, as shown in Fig. S4A, S4B, and S4C, respectively.

Statistical heterogeneity within direct comparisons and across the network was explored using the analysis of heterogeneity (ANOHE) implemented in the gemtc package under a Bayesian random-effects framework. For continuous outcomes, a normal likelihood with an identity link was used. Four Markov chains were run with 20,000 adaptation iterations and 50,000 sampling iterations. Pairwise heterogeneity was summarized using I² statistics, and pooled pairwise and network estimates were visually inspected. The global heterogeneity estimates under the consistency model were 30.2% for PNI, 59.1% for DPN, and 89.7% for CIPN.

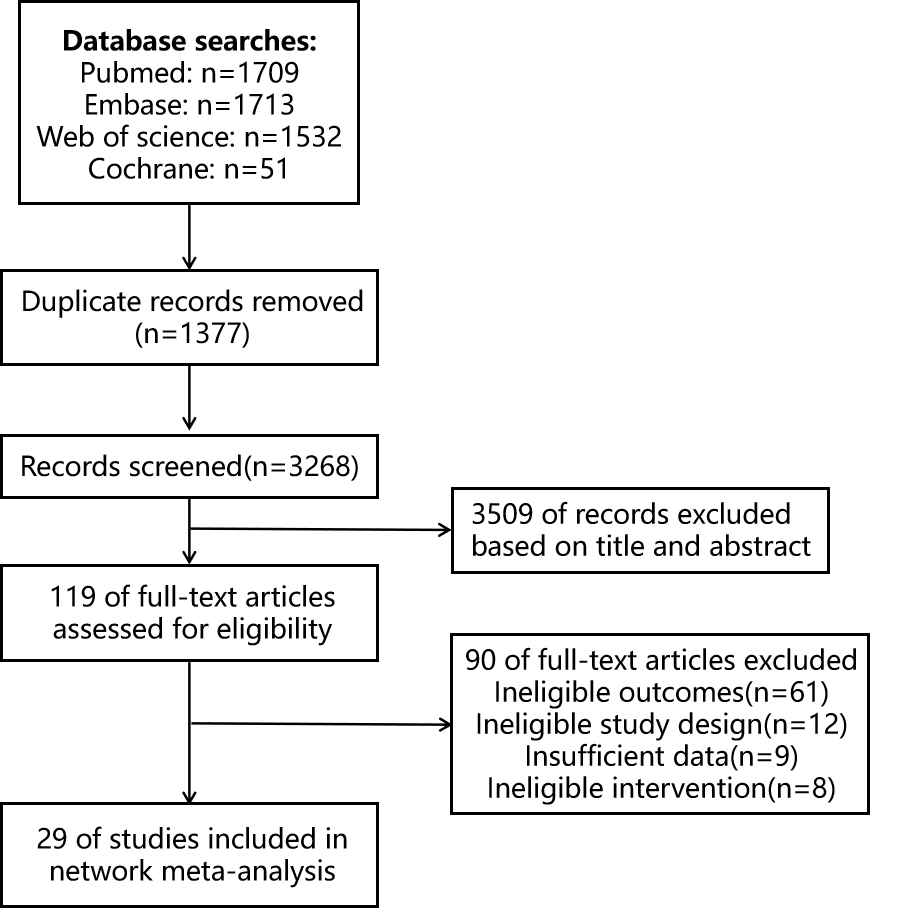

**Fig S1.** PRISMA flow diagram of study selection for the systematic review and network meta-analysis of treatments for peripheral nerve injury.


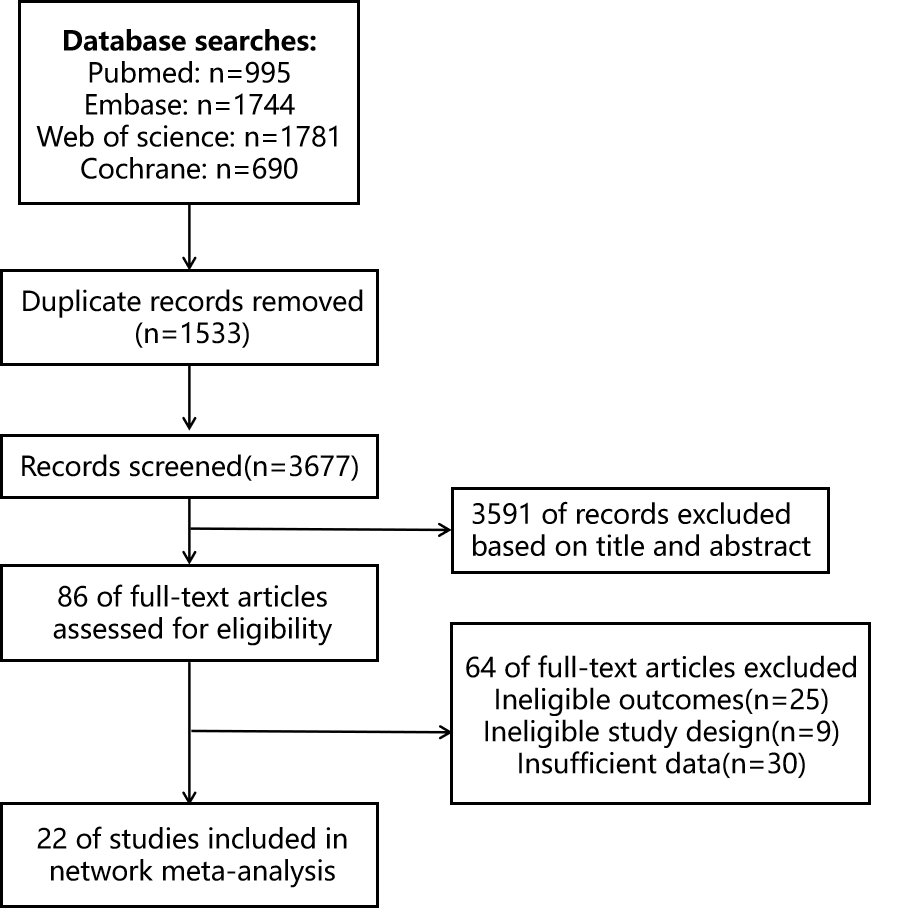

**Fig S2.** PRISMA flow diagram of study selection for the systematic review and network meta-analysis of treatments for diabetic peripheral neuropathy.


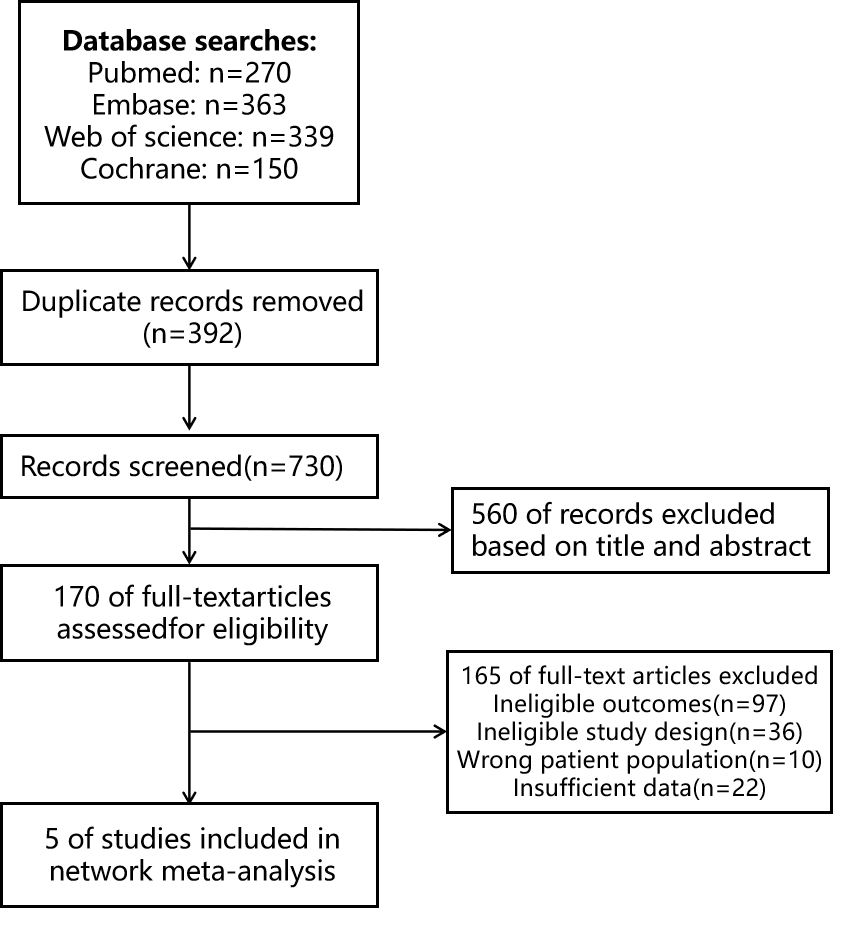

**Fig S3.** PRISMA flow diagram of study selection for the systematic review and network meta-analysis of treatments for chemotherapy-induced peripheral neuropathy.


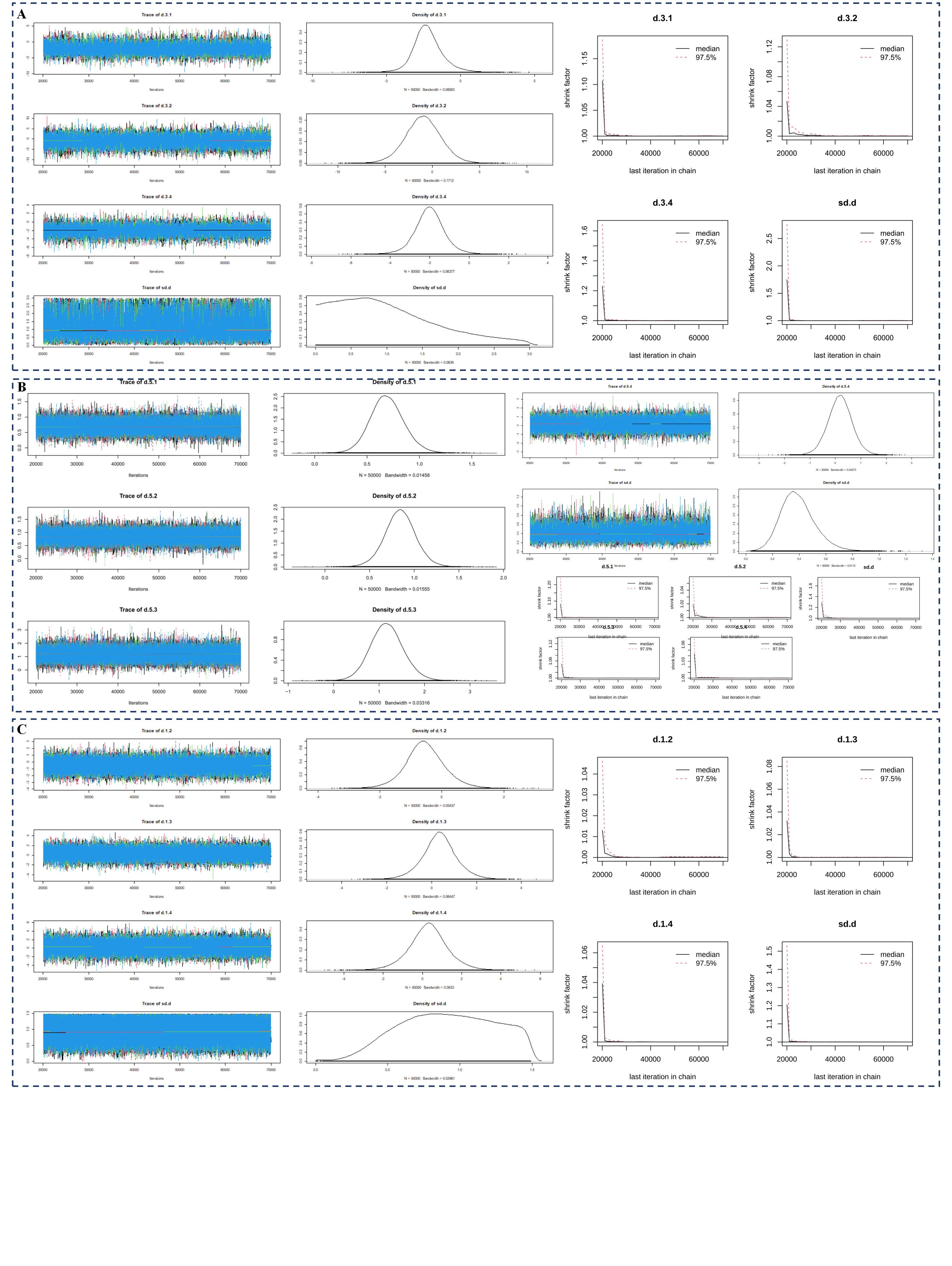

**Fig. S4.** Convergence diagnostics for the Bayesian network meta-analyses.

**2. Bibliometric**
The data analysis for this research followed a structured bibliometric and knowledge mapping approach. Data collection was initiated through a topic-specific search in the Web of Science Core Collection database, covering publications from January 1, 2001, to January 1, 2026, and restricted to document types of articles and reviews in English. The search strategy was constructed around three conceptual domains: melatonin-related terms, peripheral neuropathy-related terms, and biomaterial/tissue engineering-related terms. Representative keywords included "melatonin" and "N-acetyl-5-methoxytryptamine"; "peripheral neuropathy", "peripheral nerve injury", "diabetic peripheral neuropathy", "chemotherapy-induced peripheral neuropathy", "neuropathic pain", and "sciatic nerve injury"; as well as "tissue engineering", "biomaterial", "scaffold", "hydrogel", "nerve conduit", "controlled release", "drug delivery", "microneedle", and "nanocarrier". Following the initial retrieval, duplicate records were eliminated using the deduplication feature in CiteSpace 6.4 R1 (Advanced), yielding a final analytical corpus of 571 publications.

During the visualization stage in CiteSpace, a systematic parameter configuration was implemented. The time span was divided into 1-year slices to trace the annual evolution of research themes. A g-index (k=25) was applied for node selection to ensure representative and impactful entries were included. Multiple knowledge networks were constructed and examined: co-authorship networks to identify leading researchers and collaborative clusters; institutional and country co-occurrence networks to map geographical and organizational cooperation; and keyword co-occurrence and clustering networks to discern research focuses and thematic shifts. Key node metrics such as frequency and betweenness centrality were interpreted, and burst detection was performed to pinpoint emerging trends.

To enhance analytical depth and visual presentation, the data exported from CiteSpace, including ranked lists of high-productivity countries/regions, were imported into Tableau (version 2024.02) for further visualization processing. This step produced tailored charts such as geographic distribution maps. Collectively, the knowledge graphs generated by CiteSpace, along with the complementary visualizations created in Tableau, form the integrated visual analytical framework of this study.


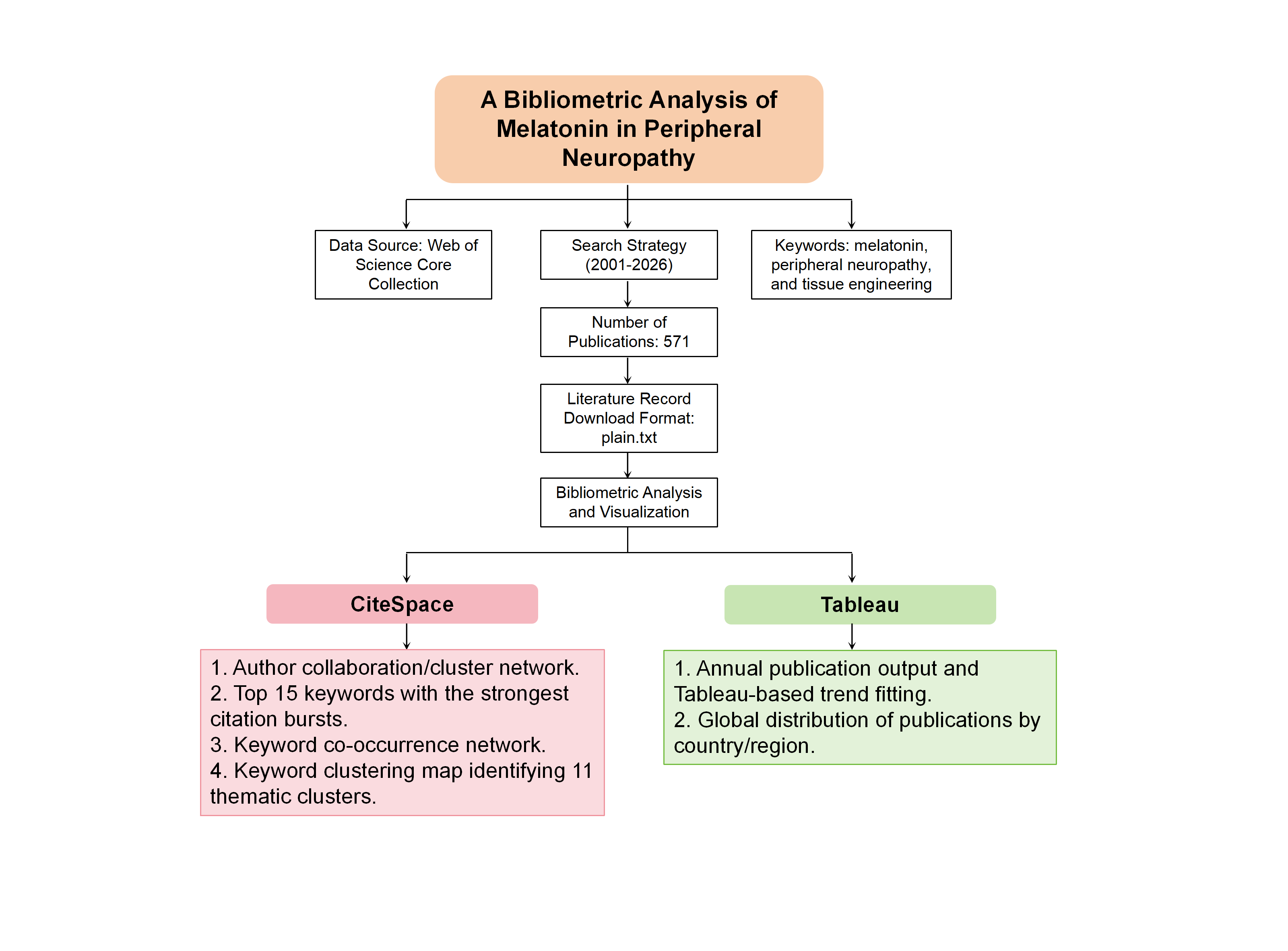
**Fig. S5.** Flowchart of the search strategy and bibliometric analysis workflow for melatonin research in peripheral neuropathy.


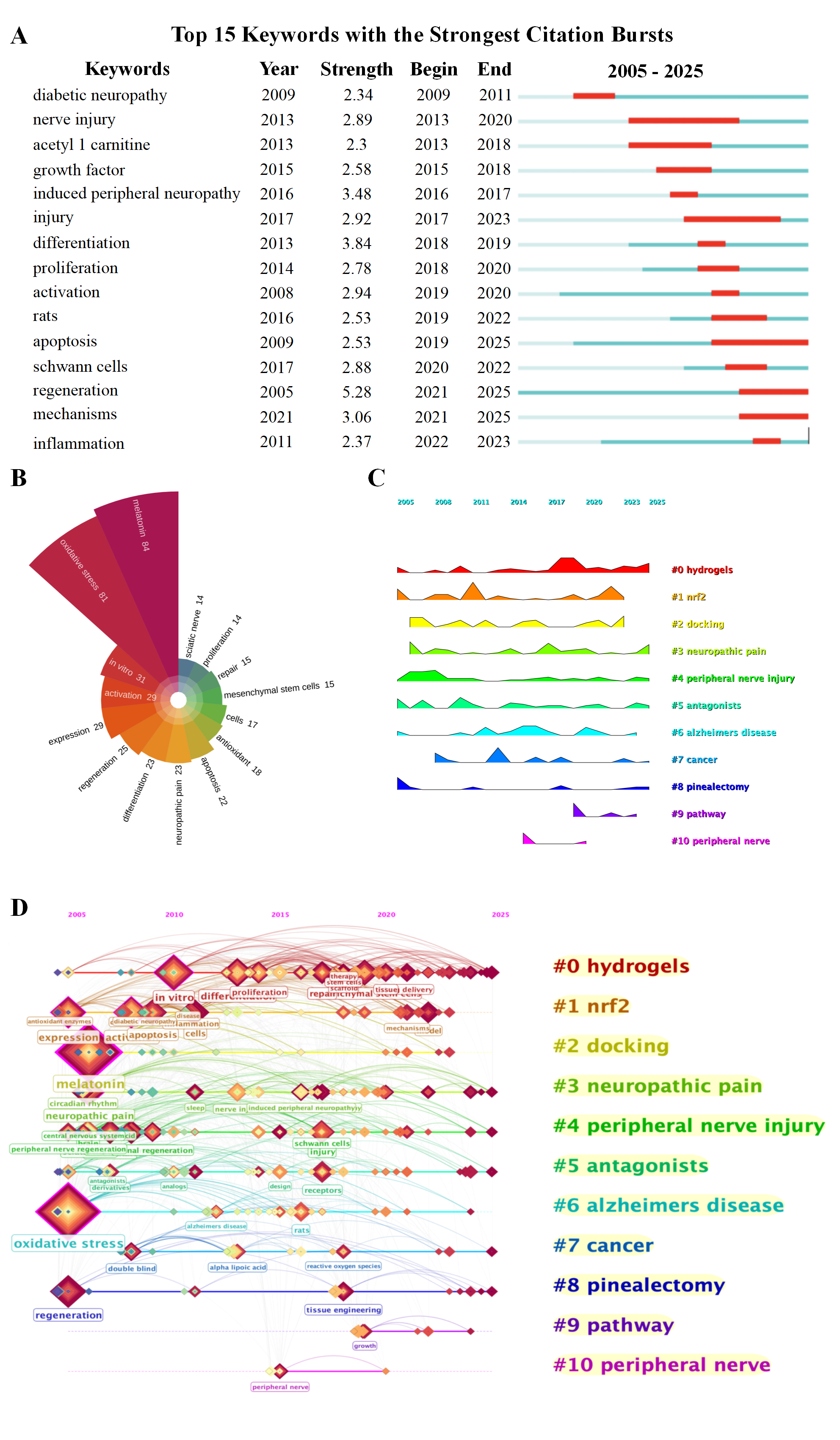

**Fig. S6.** Bibliometric analyses of melatonin-related research in peripheral neuropathy. A. Top 15 keywords with the strongest citation bursts from 2005 to 2025. B. Circular distribution of high-frequency keywords, showing the relative weights of major research terms. C. Temporal activity profiles of the 11 principal keyword clusters across the study period. D. Timeline visualization of keyword clusters depicting the emergence, persistence, and chronological evolution of major thematic domains.
